# Ultrasensitive bioorthogonal probes for selective discrimination of trace H_2_S_2_/H_2_S_*n*_ in sulfide-competitive contexts

**DOI:** 10.1016/j.apsb.2026.01.044

**Published:** 2026-02-04

**Authors:** Xidan Tong, Xiaowei Xu, Jiaxuan Chen, Jinkang Feng, Yixing Li, Yangfei Shi, Zhen Li, Weiwei Guo, Yueqin Zheng

**Affiliations:** a State Key Laboratory of Natural Medicines and Jiangsu Key Laboratory of Drug Discovery for Metabolic Diseases, Center of Drug Discovery, China Pharmaceutical University, Nanjing 211198, China; b Department of Chemistry, China Pharmaceutical University, Nanjing 211198, China; c State Key Laboratory of Natural Medicines, School of Traditional Chinese Pharmacy, China Pharmaceutical University, Nanjing 211198, China

**Keywords:** Hydrogen per/polysulfides, Fluorescent probe, Biorthogonal probe, Ultrasensitive detection, *S*-Persulfidation

## Abstract

Hydrogen persulfide/polysulfides (H_2_S_2_/H_2_S_*n*_), as an oxidized derivative of hydrogen sulfide (H_2_S), is capable of directly inducing the *S*-persulfidation of cysteine residues, thereby modulating the activity of relevant enzymes. Owing to its unique reactive properties, H_2_S_2_/H_2_S_*n*_ is emerging as a central focus in the study of reactive sulfur species. Therefore, the precise detection of H_2_S_2_/H_2_S_*n*_*in vivo* is critical for elucidating their roles in redox signaling and cellular regulation. However, conventional probes face challenges such as poor sensitivity, cross-reactivity, and instability. Here, we report a bioorthogonal ether linkage fluorescent probe toolkit (Cyne-1‒5) with a cyclooctyne warhead, enabling ultra-sensitive (LOD = 3.3 nmol/L), selective, and real-time tracking of H_2_S_2_/H_2_S_*n*_ in living systems. These probes feature rapid activation (>1018-fold fluorescence activation in 5 min), broad spectral coverage (blue to NIR), and exceptional enzymatic stability. Using this toolkit, we uncovered the spontaneous oxidation of H_2_S to trace H_2_S_2_/H_2_S_*n*_ and demonstrated steric hindrance-driven self-disproportionation of persulfides, where less bulky persulfides efficiently yield H_2_S_2_/H_2_S_*n*_. Furthermore, we achieved the cellular-level visualization of protein *S*-persulfidation dynamics. This work advances persulfide chemical biology and offers transformative tools for probing H_2_S_2_/H_2_S_*n*_ in disease mechanisms and therapeutic development.

## Graphical abstract

A bioorthogonal cyclooctyne-ether fluorescent toolkit (Cyne-1‒5) achieves selective, rapid imaging of H_2_S_2_/H_2_S_*n*_ and protein persulfidation in living systems, resolving long-standing RSS detection challenges.

**Figure:**
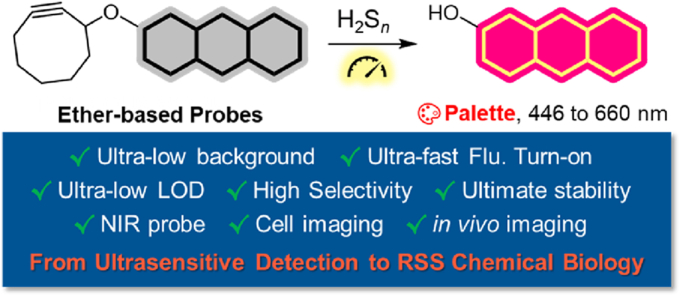

## 1 Introduction

Reactive sulfur species (RSS) constitute a diverse family of sulfur-containing molecules that play indispensable roles in regulating a broad spectrum of biological processes, particularly within redox signaling networks^1^, ^2^, ^3^, ^4^. This class includes biothiols (RSH), hydrogen sulfide (H_2_S)^5^, ^6^, ^7^, ^8^, hydrogen persulfide/polysulfides (H_2_S_2_/H_2_S_*n*_)^7^^,^^9^, ^10^, ^11^, ^12^, ^13^, hydropersulfide/polysulfides (RSS_*n*_H, *n* ≥ 1)^14^, ^15^, ^16^, and polysulfides (RSS_*n*_SR, *n* ≥ 1)^17^, ^18^, ^19^, ^20^, among others. Among these, H_2_S_2_/H_2_S_*n*_ have emerged as central mediators in redox biology due to their distinct reactivity profiles and potent signaling capabilities^21^, ^22^, ^23^, ^24^. For instance, H_2_S_2_/H_2_S_*n*_ has been shown to trigger Ca^2+^ influx in mature astrocytes at concentrations as low as 30 nmol/L, which is approximately 320-fold more potent than H_2_S^22^. This striking effect is attributed to the unique electrophilic nature of H_2_S_2_/H_2_S_*n*_, enabling direct *S*-persulfidation of protein thiols (*R*-SH to *R*-SSH), a transformation that H_2_S alone cannot efficiently mediate^25^, ^26^, ^27^, ^28^, ^29^.

These findings raise an important question: could the biological functions previously ascribed to H_2_S be mediated, at least in part, by trace amounts of H_2_S_2_/H_2_S_*n*_ present under physiological conditions? Despite this hypothesis, the *in situ* existence of H_2_S_2_/H_2_S_*n*_ in typical H_2_S donor systems has yet to be definitively confirmed. Additionally, protein persulfides (PSSH), widely considered storage forms of H_2_S *in vivo*, may liberate trace levels of H_2_S_2_/H_2_S_*n*_ during H_2_S release^3^^,^^4^. However, due to their relatively high thermodynamic stability compared to low-molecular-weight (LMW) persulfides, PSSH are unlikely to generate significant amounts of H_2_S_2_/H_2_S_*n*_ under physiological conditions. Unfortunately, these hypotheses remain speculative, largely due to the lack of highly selective and ultra-sensitive tools capable of detecting these elusive species in biological contexts. Fluorescence-based detection techniques have emerged as powerful tools for biological imaging, offering exceptional sensitivity, high spatiotemporal resolution, and non-invasive real-time monitoring^7^, ^8^, ^9^. In contrast to conventional methods such as UV‒Vis spectroscopy^21^^,^^30^, chromatography^31^, and mass spectrometry^32^^,^^33^, fluorescent probes allow for the direct visualization of analyte dynamics in living systems. Although several elegant H_2_S_2_/H_2_S_*n*_ fluorescent probes have been developed, key limitations persist^34^, ^35^, ^36^, ^37^, ^38^, ^39^, ^40^, ^41^, ^42^, ^43^, ^44^, ^45^, ^46^, ^47^, ^48^, ^49^, ^50^, ^51^, ^52^, ^53^. First, the insufficient sensitivity of current probes renders them incapable of robustly distinguishing trace H_2_S_2_/H_2_S_*n*_ (high-nmol/L to low-μmol/L) against a high background of abundant H_2_S leading to false negatives and unreliable quantification. To achieve accurate detection under these competitive conditions, a next-generation probe must possess an orders-of-magnitude higher sensitivity and affinity for H_2_S_2_/H_2_S_*n*_ over H_2_S; Second, many reported probes suffer from appreciable cross-reactivity with other RSS. Even off-target reactions that do not generate fluorescence can consume the probe, depleting its effective concentration for the intended H_2_S_2_/H_2_S_*n*_ detection and compromising quantification. Therefore, an ideal probe must exhibit not only high absolute selectivity but also superior kinetic competition efficacy, enabling it to outcompete background sulfide and react faithfully with the trace target analytes. Third, probe stability is often a neglected yet persistent concern. Labile linkers commonly used in these designs are susceptible to premature cleavage by esterases or biological media, generating background signal and compromising probe integrity before the target encounter. This underscores the need for a probe with engineered stability against non-specific hydrolysis and enzymatic interference to ensure signal fidelity in complex biological settings.

**Scheme 1:**
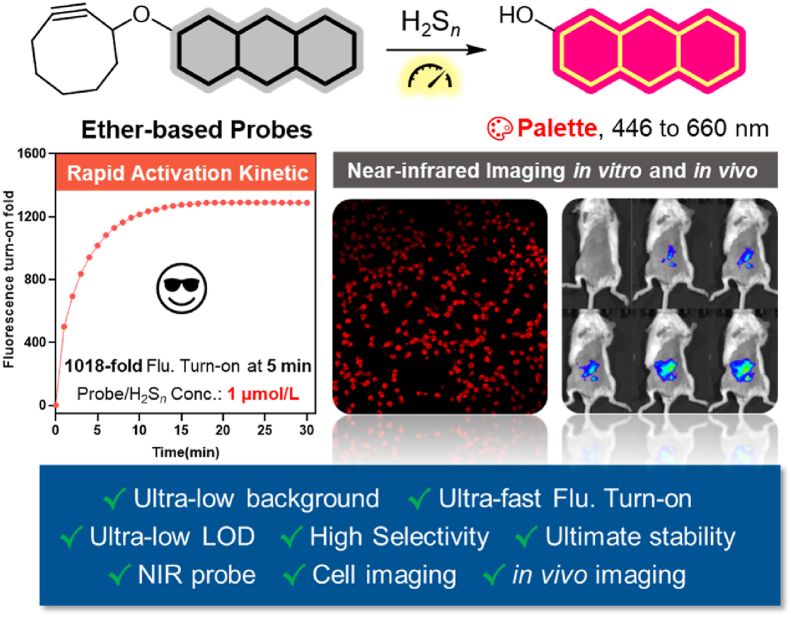
The design of cyclooctyne-based ether-linked H_2_S_2_/H_2_S_*n*_-specific fluorescent probes.

To address these longstanding challenges, we developed a series of cyclooctyne-based probes, Cyne-1‒5, in which the propargylic position of the cyclooctyne is tethered to fluorophores *via* an ether linkage. This structural design imparts significant advantages, including excellent selectivity, ultrafast fluorescence turn-on kinetics (up to 1018-fold within 5 min), an exceptionally low limit of detection (LOD = 3.3 nmol/L), and minimal background fluorescence. Notably, Cyne-1‒5 exhibit high chemo-selectivity toward H_2_S_2_/H_2_S_*n*_, making them powerful tools for the precise and sensitive detection of these species. Leveraging these advanced probes, we directly detected trace levels of H_2_S_2_/H_2_S_*n*_ in physiologically relevant H_2_S solutions and quantitatively profiled their concentrations. Furthermore, Cyne-1‒5 enabled the investigation of the intrinsic reactivity and signaling dynamics of both exogenous and endogenous persulfides, providing critical mechanistic insights into *S*-persulfidation processes *in vitro* and *in vivo* (see Scheme 1).

## 2 Results and discussion

### 2.1 Design and synthesis of the ether-based probes

**Scheme 2:**
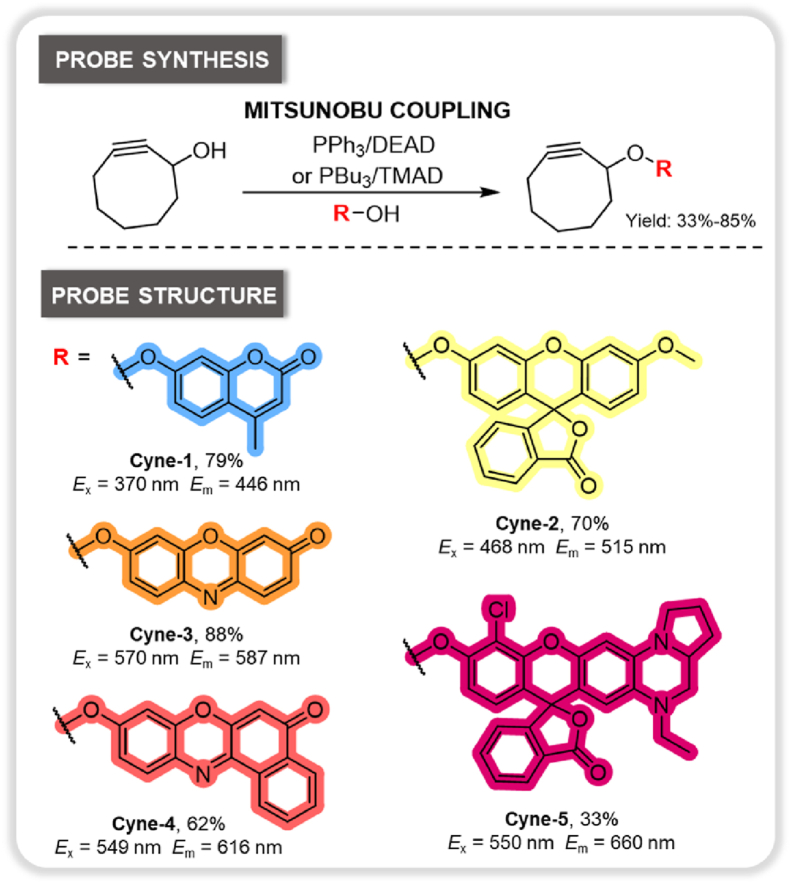
Synthesis and structure of ether linkage fluorescent probes Cyne-1‒5.

To overcome the limitations associated with ester or carbamate-based linkers—their susceptibility to enzymatic hydrolysis and their insufficient ability to effectively mask fluorophore emission in complex biological systems—we redesigned our probe architecture by replacing these moieties with an ether linkage^54^^,^^55^. Ether bonds are well-documented for their superior chemical and enzymatic stability *in vivo*. Using a one-step Mitsunobu reaction, we successfully synthesized a panel of five ether linkage fluorescent probes (Cyne-1‒5) with satisfactory yields. These probes incorporate a diverse array of fluorophores, including hydroxycoumarin, methylfluorescein^56^, resorufin, Nile red^57^, and quinoxaline rhodol (Scheme 2)^58^. This streamlined synthetic route offers considerable practical advantages: the one-step transformation is not only efficient but also operationally simple, enabling facile synthesis by researchers without extensive training in organic synthesis. This feature significantly lowers the barrier for subsequent biological and imaging studies. Importantly, the parent fluorophores span a broad emission range, including blue, yellow-green, orange, red, and near-infrared (NIR, ≥660 nm) wavelengths. This spectral diversity allows these probes to be flexibly deployed under a variety of experimental conditions, including those requiring deep-tissue penetration and minimal background autofluorescence.

### 2.2 Photophysical properties of the ether-based probes Cyne-1‒5

**Figure 1:**
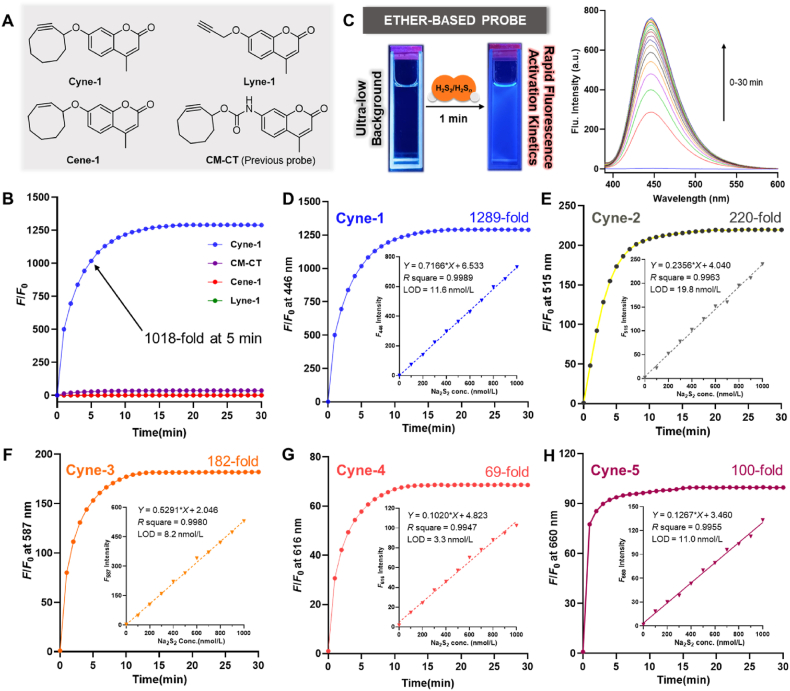
Photophysical property measurements of ether-based probe Cyne-1‒5. (A) Structures of Cyne-1, Cene-1, Lyne-1, and CM-CT. (B) Comparison of fluorescence turn-on fold of Cyne-1, Cene-1, Lyne-1 and CM-CT. The fluorescence turn-on of the hydroxycoumarin-based and aminocoumarin-based probes was monitored at *λ* = 446 nm and 470 nm, respectively. Test conditions: 1 μmol/L probe/compound with 1 μmol/L Na_2_S_2_ in PBS buffer containing 5% DMSO and 100 μmol/L CTAB. Unless otherwise specified, all the following fluorescence spectroscopic tests were performed using this solvent system. (C) Background of ether linkage probe Cyne-1 and visual comparison of its fluorescence activation. (D‒H) Time-dependent fluorescent turn-on of probes Cyne-1‒5. Inset: Concentration-dependent fluorescence turn-on of probes Cyne-1‒5.

With the synthesized probes in hand, we first evaluated their photophysical properties. Owing to the significantly suppressed intramolecular charge transfer (ICT), the fluorescence of these probes was effectively quenched, leading to a remarkably low background emission signal (Supporting Information Fig. S1). We initially investigated the reactivity of Cyne-1 with H_2_S_2_/H_2_S_*n*_. Notably, conventional H_2_S_2_/H_2_S_*n*_ probes typically require ≥100 μmol/L Na_2_S_2_ (Supporting Information Table S1), which far exceeds physiological concentrations (high-nmol/L to low-μmol/L). Such discrepancies limit their applicability in biological systems due to inadequate sensitivity and the potential need for elevated probe concentrations—raising cytotoxicity concerns. To overcome this limitation, we tested Cyne-1 at an ultra-low concentration (1 μmol/L) with an equal amount of freshly prepared Na_2_S_2_. Because H_2_S_2_/H_2_S_*n*_ are prone to rapid oxidation in ambient conditions, the reaction was carried out in ultrasonicated, deoxygenated PBS, and cuvettes were sealed post-reagent addition. Hexadecyltrimethylammonium bromide (CTAB) was included to stabilize deprotonated per/polythiolate anions (HSS_*n*_^−^, *n* ≥ 1) in aqueous solution^35^, ^36^, ^37^, ^38^, ^39^. Under these optimized conditions, Cyne-1 exhibited a rapid fluorescence turn-on—1018-fold within 5 min, eventually reaching 1289-fold (Fig. 1B‒D).

Control experiments were conducted with structural analogs Cene-1 and Lyne-1, in which the reactive cyclooctyne was replaced with *cis*-cyclooctene or a linear alkyne, respectively. Additionally, a previous-generation carbamate-based probe, CM-CT, was included for comparison (Fig. 1A)^42^. Neither Cene-1 nor Lyne-1 showed meaningful fluorescence activation, while CM-CT displayed only a modest 36-fold increase at 30 min, attributed to its high intrinsic background (Fig. 1B and C).

Next, we investigated the detection mechanism of the probe. Mechanistic studies employed the probe Cyne-*O*-PhNO_2_, engineered with a superior leaving group (*p*-nitrophenol) to achieve enhanced release kinetics (Supporting Information Fig. S2). The payload release efficiency attained 90.2% within 30 min, accompanied by complete precursor probe consumption. This outcome establishes that regioselectivity in the initial click reaction manifests through H_2_S_2_/H_2_S_*n*_ preferentially reacting with the *α*-position of the more electron-deficient alkyne rather than the *β*-position, with *β*-site engagement producing a dead-end isomer that prevents subsequent rearrangement-triggered release. Strategic implementation of benzylamine as a trapping agent successfully intercepted the critical *α*,*β*-unsaturated thioketone intermediate during the release process, with LCMS analysis clearly demonstrating diagnostic peaks corresponding to the trapped species (Supporting Information Fig. S3). These observations exhibit complete concordance with our previous research findings^42^. Additional computational studies revealed that the superior leaving group ability of the phenolic compound, compared to its aniline counterpart during the desulfurization rearrangement, thereby accelerates the fluorescence turn-on rate (Supporting Information Fig. S4).

Having thoroughly investigated the probe's detection mechanism and validated its excellent fluorescence activation kinetics, we proceeded to conduct a comprehensive spectroscopic characterization of the remaining Cyne series. Upon addition of Na_2_S_2_, each probe exhibited a clear UV‒Vis shift and rapid fluorescence turn-on (Fig. 1D–H, Supporting Information Figs. S5 and S6). In the absence of Na_2_S_2_, all probes remained highly stable for over 12 h, with negligible autoactivation (Supporting Information Fig. S7). Based on concentration-dependent fluorescence enhancement, the limit of detection (LOD) was calculated for each probe; the most sensitive, Cyne-4, achieved an LOD as low as 3.3 nmol/L (Fig. 1D–H). Remarkably, even 50 nmol/L Na_2_S_2_ induced a 7.4-fold fluorescence turn-on within 30 min (Supporting Information Fig. S8), representing among the lowest detection limits reported for H_2_S_2_/H_2_S_*n*_ fluorescent probes to date (Table S1). All probes exhibited only subtle differences in fluorescence turn-on rates, dictated by the inherent photophysical and electronic characteristics of their respective fluorophores (Table 1 and Fig. 1D–H). This consistency underscores the advantage of a highly modular and unified design: it enables the flexible development of tailored probes to meet specific experimental requirements and instrumental limitations, thereby offering significant utility in practical scenarios. Additionally, these probes exhibited robust pH tolerance, maintaining strong fluorescence turn-on across a pH range of 5.0 to 8.0 (Supporting Information Fig. S9).

**Table 1:** Spectroscopic properties of ether-based probes Cyne-1‒5 and the corresponding parent fluorophores.

Compd.	Parent fluorophore				Corresponding probe			
	*E*_x_ (nm)	*E*_m_ (nm)	*ε* (L/mol·cm)	*Φ*	*E*_x_ (nm)	*E*_m_ (nm)	*ε* (L/mol·cm)	*Φ*
Cyne-1	370	446	1.5 × 10^4^	0.36	–	–	–	–
Cyne-2	468	515	3.7 × 10^4^	0.38	–	–	–	–
Cyne-3	570	587	5.6 × 10^4^	0.74	–	–	–	–
Cyne-4	549	616	5.8 × 10^4^	0.49	–	–	–	–
Cyne-5	551	660	4.2 × 10^4^	0.20	483	595	0.8 × 10^4^	0.04

### 2.3 Selectivity toward H_2_S_2_/H_2_S_n_

**Figure 2:**
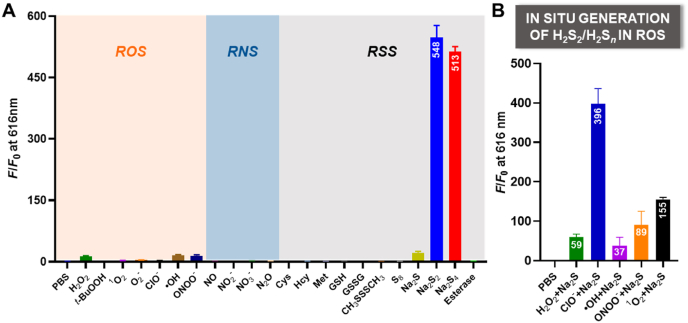
(A) *F*/*F*_0_ of probe Cyne-4 (10 μmol/L) at *E*_m_ = 616 nm in the presence of various ROS, RNS or RSS. All analytes were at 100 μmol/L concentration. (B) *F*/*F*_0_ of Cyne-4 (10 μmol/L) in the presence of ROS with H_2_S. ROS concentration: 50 μmol/L; Na_2_S concentration: 100 μmol/L. The error bars represent means ± standard deviations (SD) from three independent experiments.

To evaluate the detection selectivity, we challenged the Cyne probes with a panel of potential interferents, including reactive oxygen, nitrogen, and sulfur species (ROS, RNS, RSS), amino acids, metal ions, esterase, and representative biological media. The Cyne probes exhibited strong fluorescence enhancements exclusively in response to Na_2_S_2_ and Na_2_S_4_ (345 to 1282-fold and 345 to 1122-fold, respectively), with negligible interference from other analytes. Remarkably, the probes retained high resistance to nonspecific activation even in complex biological fluids (Fig. 2A and Supporting Information Fig. S10 and S11). This high selectivity highlights the orthogonality of the probe to physiologically abundant molecules. To mimic endogenous H_2_S_2_/H_2_S_*n*_ generation, we treated Na_2_S with various ROS. Cyne-4 alone was unresponsive to these oxidants (Fig. 2A). However, upon mixing Na_2_S with ROS, significant fluorescence increases (1.7- to 18-fold) were observed (Fig. 2B), consistent with *in situ* H_2_S_2_/H_2_S_*n*_ formation. Among all, the strongest signal resulted from NaClO, reaffirming its known efficacy in generating H_2_S_2_/H_2_S_*n*_ in biological systems^36^.

Collectively, the ether-based probe platform exhibits rapid kinetics, efficient payload release, ultra-low detection limits, exceptional selectivity, and broad spectral coverage, making it ideally suited for sensitive H_2_S_2_/H_2_S_*n*_ detection.

### 2.4 Trace detection of H_2_S_2_/H_2_S_n_ in Na_2_S solution

Interestingly, a weak but consistent fluorescence increase was observed when Cyne-4 was incubated with Na_2_S solution, suggesting potential oxidation to trace H_2_S_2_/H_2_S_*n*_. Given the previously confirmed stability of the probe (Fig. S7), we hypothesized that trace H_2_S_2_/H_2_S_*n*_ formation accounts for this signal.

**Figure 3:**
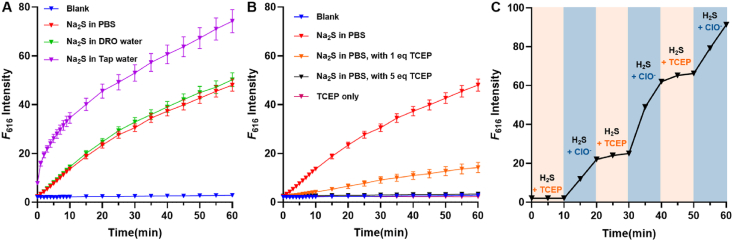
(A) Time-dependent curve of fluorescence intensity changes of Na_2_S in different solvents. (B) Time-dependent curve of fluorescence intensity changes of Na_2_S after the addition of different equivalents of TCEP. (C) Time-dependent curve of fluorescence intensity changes when TCEP and NaClO are alternately added. Test conditions: probe Cyne-4: 10 μmol/L; Na_2_S: 100 μmol/mol; TCEP: 100 μmol/L (1 eq.) or 500 μmol/L (5 eq.); NaClO: 500 μmol/L. The concentration of the NaClO solution is calibrated *via* iodometry prior to each fluorescence test. The error bars represent means±standard deviations (SD) from three independent experiments.

To explore this, we prepared Na_2_S in PBS, double reverse osmosis deionized water (DRO water), and municipal tap water. In all cases, fluorescence turn-on was detected, with similar intensities in PBS and DRO water (20.7- and 21.0-fold at 60 min), and higher signal in tap water (30.8-fold), attributed to hypochlorite-mediated oxidation (Fig. 3A). For comparison, Na_2_S_2_ triggered a 427-fold fluorescence enhancement within 1 min, indicating that the low response from Na_2_S does not impair probe specificity (Supporting Information Fig. S12). Based on fluorescence intensities, the proportion of H_2_S_2_/H_2_S_*n*_ generated from Na_2_S oxidation was estimated at 0.43%–0.48% per hour (Supporting Information Fig. S13).

We further validated this by adding tris(2-carboxyethyl) phosphine (TCEP), a water-soluble reductant that prevents H_2_S oxidation and reverses disulfide formation^23^. TCEP alone neither activated fluorescence nor consumed the probe (Fig. 3B and C). Co-treatment with TCEP suppressed Na_2_S-induced fluorescence, and increasing TCEP fully abolished the signal. In a “switch” experiment, alternate addition of TCEP and NaClO modulated fluorescence in an “off–on–off” pattern (Fig. 3C), confirming that the signal originated from H_2_S_2_/H_2_S_*n*_.

These results conclusively demonstrate that Na_2_S solution undergo slow oxidation to trace H_2_S_2_/H_2_S_*n*_, and that our probes are sufficiently sensitive and selective to detect this process. These findings raise important questions about whether such low-level species may contribute to the biological effects previously attributed solely to H_2_S.

### 2.5 Imaging and characterization of exogenous H_2_S_2_/H_2_S_n_ in living cells

**Figure 4:**
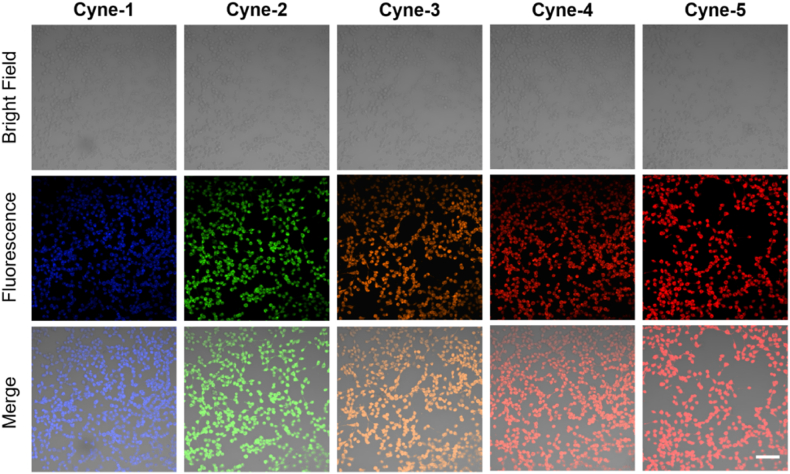
Confocal microscopy images of the probe Cyne family in response to exogenous H_2_S_2_/H_2_S_*n*_ addition in 4T1 cells. 4T1 cells were incubated with the probes (2 μmol/L) for 30 min, then washed by PBS buffer and treated with Na_2_S_2_ (10 μmol/L). Images were acquired after 15 min. Scale bar represents 100 μm in all images.

We next evaluated the potential of the Cyne probe series for intracellular imaging of H_2_S_2_/H_2_S_*n*_. To assess their ability to visualize exogenous H_2_S_2_/H_2_S_*n*_ in live cells, 4T1 cells were incubated with each probe (2 μmol/L) for 30 min, followed by washing to remove excess extracellular probe. Subsequently, Na_2_S_2_ (10 μmol/L) was added and incubated for an additional 15 min prior to imaging with confocal laser scanning microscopy (CLSM). As shown in Fig. 4, all Cyne probes produced strong intracellular fluorescence in response to Na_2_S_2_ treatment, indicating effective activation and cellular uptake. In contrast, cells not treated with Na_2_S_2_ or those exposed to other biologically relevant sulfur species—such as glutathione (GSH), cysteine (Cys), cyclooctasulfur (S_8_), or Na_2_S—exhibited negligible fluorescence signals (Supporting Information Fig. S14), highlighting the excellent selectivity of the probes in cellular environments. The observed membrane permeability is likely due to the lipophilic nature of the cyclooctyne moiety, facilitating efficient intracellular delivery. Importantly, cytotoxicity assays confirmed that the Cyne probes exhibit minimal toxicity in 4T1 cells, with no significant reduction in cell viability observed at concentrations up to 50 μmol/L (Supporting Information Fig. S15), underscoring their suitability for biological imaging applications.

### 2.6 Monitoring H_2_S-derived H_2_S_2_/H_2_S_n_ formation in cells *via* ROS-mediated oxidation

Our previous results revealed that trace amounts of H_2_S_2_/H_2_S_*n*_ can form *via* oxidation in aqueous H_2_S solutions. This observation prompted us to ask: does a similar phenomenon occur within cells or tissues? This question is particularly relevant for understanding the physiological effects of H_2_S-releasing prodrugs, especially those designed to generate H_2_S intracellularly. If endogenous or prodrug-derived H_2_S can give rise to H_2_S_2_/H_2_S_*n*_ in the presence of cellular ROS, these species may play previously unrecognized roles in mediating biological responses.

**Figure 5:**
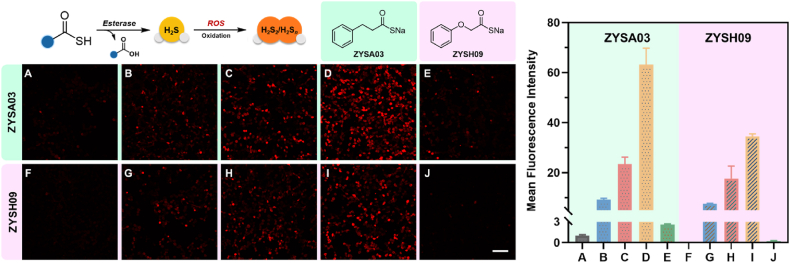
Left: Confocal microscopy images of H_2_S generated from thiocarboxylic acid reacting with various ROS to form H_2_S_2_/H_2_S_*n*_*in situ* in 4T1 cells. 4T1 cells were incubated with the probe Cyne-5 (5 μmol/L) for 30 min, then washed by PBS and then treated with thiocarboxylic acids (ZYSH03 or ZYSH09, 50 μmol/L) for another 30 min. After being washed with PBS, the cells were treated with (A, F) vehicle; (B, G) 50 μmol/L of H_2_O_2_; (C, H) 500 μmol/L of H_2_O_2_; (D, I) 50 μmol/L of ClO^‒^ or (E, J) 50 μmol/L of ClO^‒^, but the cells were pre-treated with the esterase inhibitor PMSF before the addition of thiocarboxylic acid in the previous step. Images were acquired after 30 min. Scale bar represents 100 μm in all images. Right: Quantitative graph of fluorescence intensities of the probe Cyne-5 staining. Data are mean ± standard deviations (SD) calculated using ImageJ software (*n* = 3).

Given the high sensitivity and selectivity of our ether-based probes, we sought to directly monitor the intracellular generation of H_2_S_2_/H_2_S_*n*_ from endogenous H_2_S produced *in situ*. Specifically, we aimed to evaluate the differences in H_2_S_2_/H_2_S_*n*_ production rates when cells were exposed to various ROS. Recently, our group developed a class of thiocarboxylic acid-based H_2_S donors that are both hydrolytically stable and capable of releasing H_2_S rapidly upon esterase activation (Fig. 5)^59^. The rate of H_2_S release is tunable based on the structural features of the thiocarboxylic acid core. These donors display excellent biocompatibility and favorable pharmacokinetic profiles. A major advantage of this system lies in its ability to generate H_2_S in a sustained, intracellular manner, avoiding the cytotoxicity often associated with bolus administration of high H_2_S concentrations and reducing the risk of rapid diffusion and loss before interaction with ROS.

To explore intracellular H_2_S_2_/H_2_S_*n*_ formation, we selected two structurally distinct donors—ZYSA02 and ZYSH09—for evaluation. 4T1 cells were treated with either donor (50 μmol/L) alone or in combination with various ROS, followed by imaging with our fluorescent probes. Donor-only treatment elicited minimal fluorescence (entries A, F), confirming the probes’ selectivity. However, significant fluorescence turn-on was observed upon ROS co-treatment (entries B–D, G–I), with the extent of activation depending on the type and concentration of ROS. Low concentrations of H_2_O_2_ (50 μmol/L) induced modest fluorescence increases (entries B, G), while ClO^−^ at the same concentration resulted in markedly stronger activation (entries D, I). Increasing the H_2_O_2_ concentration to 500 μmol/L enhanced the signal (entries C, H), though the fluorescence intensity remained lower than that triggered by ClO^−^ at 50 μmol/L. These results suggest that ClO^−^ is significantly more efficient at converting H_2_S into H_2_S_2_/H_2_S_*n*_ than H_2_O_2_ under physiological conditions. To confirm that fluorescence activation originated from H_2_S release, cells were pre-treated with phenylmethylsulfonyl fluoride (PMSF), an esterase inhibitor^60^. PMSF treatment abolished fluorescence even in the presence of ClO^−^, demonstrating that donor hydrolysis—and thus H_2_S generation—is a prerequisite for H_2_S_2_/H_2_S_*n*_ formation under these conditions. Interestingly, ZYSA02 and ZYSH09 exhibited different fluorescence responses under identical conditions, consistent with their distinct H_2_S release kinetics (Supporting Information Table S2). This highlights the capability of our probe system to discern subtle differences in intracellular H_2_S dynamics and oxidative conversion efficiencies.

Collectively, these results provide compelling evidence that intracellularly generated H_2_S can be oxidized by ROS to yield H_2_S_2_/H_2_S_*n*_, and that the efficiency of this conversion depends critically on both ROS identity and the kinetics of H_2_S release. Notably, these findings align with earlier studies by the Wang group, which demonstrated that ClO^−^ reacts with chalcogen species within seconds, whereas H_2_O_2_ requires hundreds of hours to reach comparable oxidation levels^61^, ^62^, ^63^. This substantial rate disparity underscores the importance of ROS type in modulating H_2_S_2_/H_2_S_*n*_ signaling in biological systems.

### 2.7 Quantitative detection of endogenous H_2_S_2_/H_2_S_n_ production in cells

**Figure 6:**
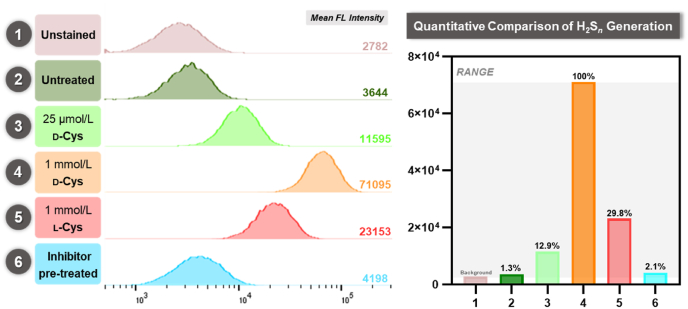
Detection of endogenous H_2_S_2_/H_2_S_*n*_ in flow cytometry with the probe Cyne-2. In a six-well plate, HEK293T cells were incubated with the probe Cyne-2 (2 μmol/L) for 30 min, then washed and subjected to different treatments. 1) Unstained; 2) Cells were incubated with FBS-free DMEM for 30 min; 3) Cells were incubated with d-cysteine (25 μmol/L) for 30 min; 4) Cells were incubated with d-cysteine (1 mmol/L) for 30 min; 5) Cells were incubated with L-cysteine (1 mmol/L) for 30 min; 6) Cells were pre-incubated with the inhibitor sodium benzoate (500 μmol/L) for 30 min, then washed and incubated with the probe Cyne-2 (2 μmol/L) for 30 min, followed by washing and incubation with d-cysteine (1 mmol/L) for 30 min. Subsequently, samples were standardized and analysed using flow cytometry. *E*_x_ wavelength: 488 nm; *E*_m_ channel: 530/30 nm. Results are representative of three independent experiments.

After confirming the robust performance of our probes for imaging exogenous H_2_S_2_/H_2_S_*n*_ in live cells, we next sought to evaluate their capability in tracking the production of endogenous H_2_S_2_/H_2_S_*n*_. Previous studies have demonstrated that d-cysteine (D-Cys) stimulation in renal cells constitutes an effective model system for endogenous H_2_S_2_/H_2_S_*n*_ generation^64^. This established platform is further employed to validate the sensitivity of probes toward endogenous H_2_S_2_/H_2_S_*n*_^36^. Here, we employed flow cytometry for providing a detailed view of dynamic H_2_S_2_/H_2_S_*n*_ generation across a cell population. In HEK293T cells, the probe Cyne-2 exhibited minimal basal fluorescence, confirming its low background signal (Fig. 6, entry 2). Upon treatment with 25 μmol/L D-Cys, a 3.2-fold increase in fluorescence was observed (Fig. 6, entry 3), indicating initial endogenous H_2_S_2_/H_2_S_*n*_ formation. When the D-Cys concentration was increased to 1 mmol/L, the fluorescence signal further surged to 19.6-fold over baseline (Fig. 6, entry 4), suggesting significant enhancement of H_2_S_2_/H_2_S_*n*_ production under these conditions. To verify that this effect was dependent on DAO-mediated metabolism of D-Cys, we pretreated cells with sodium benzoate, a known DAO inhibitor. Under this condition, even 1 mmol D-Cys failed to elicit fluorescence activation (Fig. 6, entry 6), confirming that DAO activity is essential for the observed H_2_S_2_/H_2_S_*n*_ production. Interestingly, L-cysteine (1 mmol/L) also triggered a modest fluorescence increase (Fig. 6, entry 5); however, the magnitude was only 29.8% of that observed with D-Cys under the same conditions. This result supports previous findings from the Kimura group, which demonstrated that under physiological pH (7.4), D-Cys is significantly more effective than L-Cys in inducing the endogenous production of H_2_S_2_/H_2_S_*n*_, particularly in renal and neural tissues. Collectively, these results highlight the utility of our probes in tracking real-time, endogenous H_2_S_2_/H_2_S_*n*_ fluctuations with high sensitivity and cellular resolution. Furthermore, the ability to distinguish between D-Cys and L-Cys pathways reinforces the biological relevance of stereospecific enzymatic regulation in sulfur metabolism.

### 2.8 *In vivo* imaging of exogenous and endogenous H_2_S_2_/H_2_S_n_ in BALB/c mice

**Figure 7:**
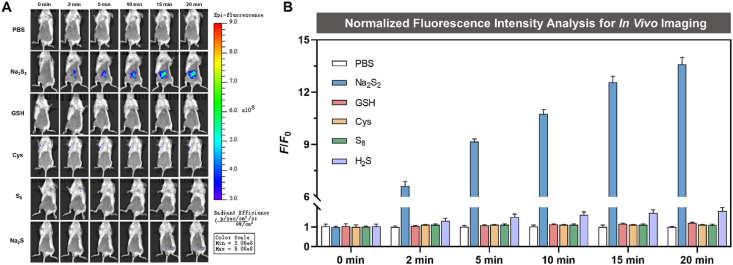
*In vivo* imaging of H_2_S_2_/H_2_S_*n*_ in BALB/c mice. (A) Representative images of the probe Cyne-5 specificity for detecting H_2_S_2_/H_2_S_*n*_ in live BALB/c mice. Each mouse was intraperitoneally administered 100 μL of the probe Cyne-5 (10 μmol/L in PBS buffer, containing 0.1% DMSO). Fifteen min later, the mice were each injected with 100 μL of PBS buffer/Na_2_S_2_ (1 mmol/L)/GSH (1 mmol/L)/Cysteine (1 mmol/L)/S_8_ (1 mmol/L, containing 1% EtOH)/Na_2_S (1 mmol/L). Imaging was performed using the PerkinElmer IVIS® Lumina LT Series III *in vivo* imaging system. Excitation filter: 570 nm; Emission filter: 660 nm. (B) Quantitative analysis of Cyne-5 staining fluorescence intensity. Data are mean values ± standard deviations (SD) calculated using LivingImage® software (*n* = 3).

We first assessed the ability of Cyne-5 to detect exogenous H_2_S_2_/H_2_S_*n*_ in BALB/c mice. Mice (*n* = 3 per group) received an intraperitoneal injection of Cyne-5, followed 15 min later by PBS, Na_2_S_2_, GSH, Cys, S_8_, or Na_2_S. Whole-body fluorescence imaging was performed using the PerkinElmer IVIS® Lumina LT Series III *in vivo* imaging system. As shown in Fig. 7, the addition of Na_2_S_2_ elicited a rapid and significant fluorescence enhancement within 2 min, which plateaued at approximately 20 min and remained stably detectable for over 60 min (Supporting Information Fig. S17). In contrast, negligible fluorescence was observed in the other groups, confirming the high selectivity of Cyne-5 *in vivo*. Meanwhile, pharmacokinetic analysis of the plasma concentration in mice revealed that the blood level peaked approximately 20 min after intraperitoneal injection, followed by rapid clearance. This kinetic profile suggests a low potential for accumulation-related toxicity *in vivo* (Supporting Information Fig. S18).

We then investigated the probe's ability to visualize endogenously generated H_2_S_2_/H_2_S_*n*_. Previous studies have demonstrated that lipopolysaccharide (LPS)-induced inflammation can stimulate endogenous production of H_2_S_2_/H_2_S_*n*_, providing a well-established model. BALB/c mice were divided into two groups (*n* = 3): one received LPS, the other saline. After 24 h, Cyne-5 was administered intraperitoneally, followed by imaging. As shown in Supporting Information Fig. S16, the LPS-treated group exhibited strong, time-dependent fluorescence, while the saline control group showed minimal signal.

Together, these results confirm that Cyne-5 enables rapid, selective, and real-time visualization of both exogenous and endogenous H_2_S_2_/H_2_S_*n*_ *in vivo*, highlighting its strong potential for biological and pathophysiological applications.

### 2.9 Exploring the reactivity of persulfides with varying steric hindrance

Protein *S*-persulfidation (*R*-SH to *R*-SSH) has emerged as an important post-translational modification, modulating the activity of numerous signaling proteins, such as the inactivation of GAPDH^4^^,^^11^^,^^25^^,^^26^. While H_2_S is a key mediator of persulfidation, it cannot directly modify protein thiols due to its redox state. Instead, protein thiols are first oxidized to sulfenic acids (P-SOH), which then react with H_2_S to form P-SSH. P-SSH can be further oxidized but uniquely possesses the ability to revert to thiols *via* sulfane sulfur sacrifice, thus providing a redox-buffering function. Low molecular weight persulfides (LMW-RSSH), such as Cys-SSH and GSSH, can also transfer their sulfhydryl sulfur to protein thiols *via* transpersulfidation^4^.

Owing to their low p*K*_a_ and *α*-effect, LMW-RSSH primarily exist as RSS^−^, exhibiting enhanced nucleophilic character^65^^,^^66^. However, within specific microenvironments *in vivo*, persulfides may also exist in their protonated form, thereby functioning as electrophilic species^33^^,^^67^, ^68^, ^69^. Thiols lack this unique property and are generally considered solely nucleophilic. Both sulfur atoms in persulfides possess electrophilic character, the fate of persulfides with thiols—whether undergoing disulfide formation, H_2_S release, or transpersulfidation—is influenced by their steric hindrance, as recently demonstrated by the Pluth group^69^. Bulky persulfides favor transpersulfidation, while less bulky ones tend to form disulfides and release H_2_S.

**Figure 8:**
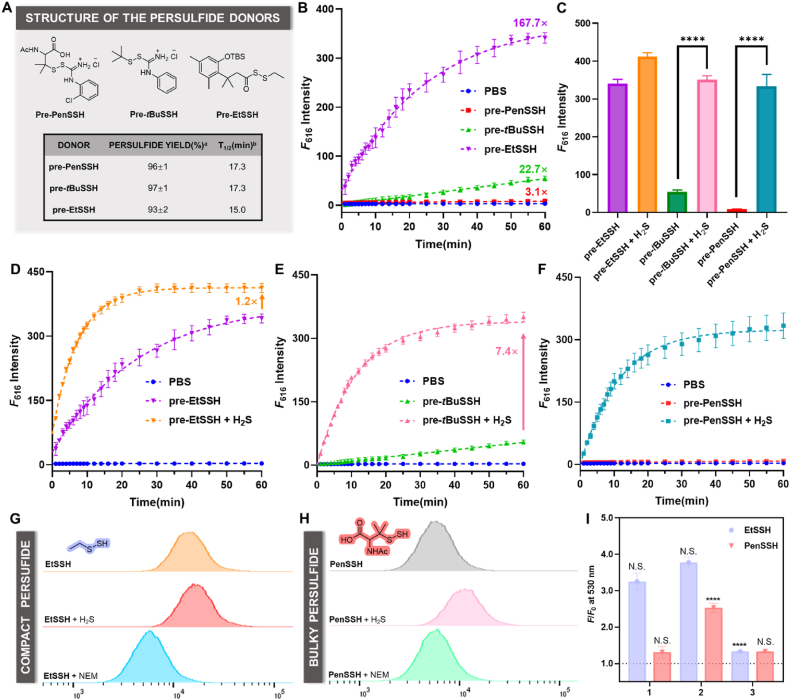
(A) Chemical structures of persulfide donors Pre-PenSSH, Pre-*t*BuSSH and Pre-EtSSH. They have high payload release rates and similar half-lives (See inset, ^a^ Corresponding RSSH yield was calculated based upon % donor consumed. ^b^Reported data are detailed in Refs. 42 and 70). (B) Fluorescent time-dependent activation of H_2_S_2_ generation *via* persulfides disproportionation. (C‒F) Comparison of fluorescence activation by persulfides disproportionation in the presence or absence of H_2_S. Test conditions: probe Cyne-4: 10 μmol/L; persulfide donors: 100 μmol/L; Na_2_S: 100 μmol/L. (G, H) Comparison of persulfides with different steric hindrance in the presence or absence of H_2_S or NEM in the generation of H_2_S_2_/H_2_S_*n*_ by flow cytometry. (I) Fold change in fluorescence intensity (*F*/*F*_0_) compared to the untreated group. In a six-well plate, 4T1 cells were incubated with the probe Cyne-2 (2 μmol/L) for 30 min, then washed and subjected to different treatments. 1) Only the persulfide donors (20 μmol/L) were added, followed by the corresponding trigger; 2) Based on the former, Na_2_S (20 μmol/L) was finally added; 3) Similar to treatment 2, but NEM (100 μmol/L) was pre-incubated before the addition of the persulfide donors. Subsequently, samples were standardized and analyzed using flow cytometry. *E*_x_ wavelength: 488 nm; *E*_m_ channel: 530/30 nm. Results are representative of three independent experiments. ∗∗∗∗*P* < 0.0001.

We hypothesized that the self-disproportionation of persulfides also depends on steric bulk, with less bulky persulfides favoring H_2_S_2_/H_2_S_*n*_ formation. To test this, we synthesized three donors—Pre-PenSSH, Pre-*t*BuSSH, and Pre-EtSSH—precursors of persulfides with decreasing bulkiness (Fig. 8A and Supporting Information Fig. S19)^42^^,^^70^. All three showed comparable stability and half-lives, and no fluorescence activation in the absence of trigger (Supporting Information Fig. S20). Upon triggered, they yielded increasing fluorescence with Cyne-4: 3.1-fold (PenSSH), 22.7-fold (*t*BuSSH), and 167.7-fold (EtSSH), indicating higher H_2_S_2_/H_2_S_*n*_ formation from less hindered persulfides (Fig. 8B).

To assess whether H_2_S enhances disproportionation, we pre-incubated each donor with H_2_S before triggering. Without triggered, fluorescence remained low (Supporting Information Fig. S21). Upon triggering, co-treatment with H_2_S increased fluorescence by 46.6-fold (PenSSH), 7.4-fold (*t*BuSSH), and 1.2-fold (EtSSH) compared to no-H_2_S controls (Fig. 8C–F). This suggests that H_2_S can accelerate transpersulfidation and H_2_S_2_/H_2_S_*n*_ release, particularly in bulky persulfides, which are otherwise relatively stable.

We further validated this in 4T1 cells using flow cytometry. After the probe loading, cells were treated with Pre-PenSSH or Pre-EtSSH (20 μmol/L) with or without Na_2_S. EtSSH alone induced a 3.3-fold fluorescence increase, while PenSSH showed minimal activation (1.3-fold). Upon H_2_S addition, PenSSH activation increased significantly (2.6-fold), whereas EtSSH showed little further enhancement. Pre-treatment with NEM, a thiol scavenger, abolished signal in both groups (Fig. 8G‒I and Supporting Information Fig. S22 and S23)^29^.

Based on the aforementioned results, we propose that the observed divergence in persulfide self-disproportionation kinetics is primarily sterically controlled. Although the sulfenyl sulfur (RSSH) of the persulfide moiety exhibits stronger electrophilicity, its susceptibility to nucleophilic attack is significantly attenuated by substantial steric hindrance, particularly in bulky persulfide species. In contrast, the sulfhydryl sulfur (RSSH), while sterically unencumbered, maintains a dynamic nucleophilic/electrophilic equilibrium due to protonation/deprotonation equilibria, resulting in comparatively weaker electrophilicity than the sulfenyl sulfur. Consequently, sterically hindered persulfides demonstrate markedly enhanced stability over their less hindered counterparts, exhibiting minimal propensity for self-disproportionation. Conversely, low-steric-hindrance persulfides undergo rapid degradation to generate H_2_S_2_/H_2_S_*n*_. Notably, when exposed to H_2_S (the smallest thiol species), the attack selectivity is substantially diminished, enabling all persulfide variants to rapidly produce H_2_S_2_/H_2_S_*n*_ regardless of their steric profiles.

### 2.10 Real-time monitoring of intracellular protein S-persulfidation levels

The Tag-Switch method, although widely employed for the detection of protein *S*-persulfidation, primarily functions as an end-point analytical assay and thus cannot capture the dynamic changes of *S*-persulfidation in living systems^71^, ^72^, ^73^. Moreover, it requires relatively complex sample processing. Building on our previous finding that persulfides undergo disproportionation to generate H_2_S_2_/H_2_S_*n*_, we propose that the levels of H_2_S_2_/H_2_S_*n*_ can serve as reliable surrogates for overall protein *S*-persulfidation. This fluorescence-based approach offers a complementary means to the Tag-Switch assay, enabling indirect yet robust, real-time monitoring of global *S*-persulfidation dynamics in living systems, while the Tag-Switch method provides molecular specificity and protein-level identification.

**Figure 9:**
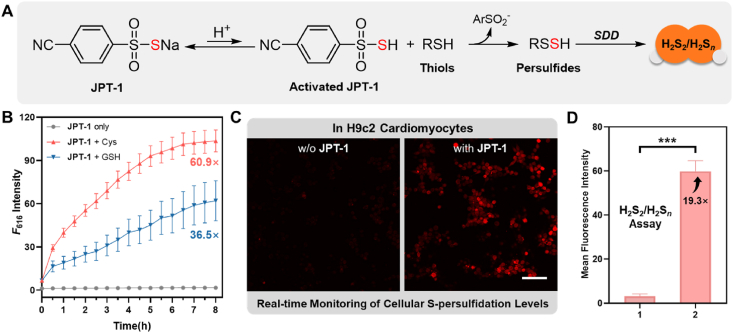
Tracking the dynamics of intracellular RSSH levels through the fluctuations of H_2_S_2_/H_2_S_*n*_. (A) Mechanism of RSSH and H_2_S_2_/H_2_S_*n*_ generation from donor JPT-1. SDD = Self-disproportionation degradation. (B) Fluorescence turn-on response of JPT-1 with or without biothiols. Test condition: Cyne-5: 5 μmol/L; JPT-1: 100 μmol/L; biothiols (Cys/GSH): 2 mmol/L. (C) Confocal microscopy images of fluctuations in H_2_S_2_/H_2_S_*n*_ levels in H9c2 cardiomyocytes under different treatments. H9c2 cardiomyocytes were incubated with 1) FBS-free DMEM or 2) JPT-1 (1 mmol/L) for 24 h. Then, after washing by PBS, the cells were subjected to Cyne-5 (5 μmol/L). Images were acquired after 30 min. Scale bar represents 100 μm in all images. (D) Quantitative graph of fluorescence intensities of the probe Cyne-5 staining in Fig. 9C. Data are mean values ± standard deviations (SD) calculated using ImageJ software (*n* = 3). ∗∗∗*P* < 0.001.

Recently, the Toscano group reported an exogenous or endogenous thiol-activated persulfide donor, JPT-1, which was demonstrated to be an effective tool for elevating intracellular RSSH levels (Fig. 9A)^74^. We sought to determine whether our probe could mimic the changes in protein *S*-persulfidation levels during this process. Incubation of the donor JPT-1 (100 μmol/L) with Cyne-5 (5 μmol/L) for 8 h did not induce any fluorescence turn-on. However, upon addition of biological thiols (2 mmol/L) to the reaction mixture, a significant fluorescence enhancement was observed. Treatment with Cys and GSH increased the fluorescence intensity by 60.9- and 36.5-fold, respectively (Fig. 9B). This result indicates that our probe can specifically detect fluctuations in H_2_S_2_/H_2_S_*n*_ levels during this process. Subsequently, we further validated the probe in H9c2 cardiomyocytes. Cells treated with JPT-1 exhibited a marked fluorescence increase, reaching 19.3-fold higher than that of the control group (Fig. 9C and D). This result highlights the potential of this probe as a tool for monitoring fluctuations in intracellular RSSH levels. The application of this probe, as a complement to the Tag-Switch technology, provides a novel method and tool for real-time dynamic monitoring of protein *S*-persulfidation levels in living systems.

## 3 Conclusions

In this work, we have developed a novel class of ether-based fluorescent probes (Cyne-1‒5) featuring a bioorthogonal cyclooctyne motif for the real-time, ultra-sensitive, and highly selective detection of H_2_S_2_/H_2_S_*n*_ *in vitro* and *in vivo*. The ether linkage, in contrast to earlier carbamate-based architectures, offers markedly improved reactivity, sensitivity, and stability, while significantly minimizing background fluorescence. The Cyne probes exhibit broad NIR fluorescence coverage (>660 nm), ultra-fast response kinetics (up to 1018-fold turn-on within 5 min), and trace-level detection sensitivity (LOD down to 3.3 nmol/L), along with high selectivity against other ROS, RNS, and RSS.

Using these probes, we provided the first direct evidence that H_2_S can spontaneously oxidize to form trace H_2_S_2_/H_2_S_*n*_ under physiological conditions, highlighting the broader biological relevance of these species. This toolkit enables unprecedented access to the chemical biology of persulfides, including their degradation behavior, reactivity, and role in redox signaling.

We systematically investigated the self-disproportionation of persulfides and established that steric hindrance critically governs their reactivity. Persulfides with small steric hindrance readily produce H_2_S_2_/H_2_S_*n*_, while bulky species are less reactive unless co-treated with H_2_S, which accelerates H_2_S_2_/H_2_S_*n*_ formation *via* transpersulfidation. To our knowledge, this is the first study to delineate the steric effects governing persulfide degradation pathways. Importantly, we established a novel, non-invasive approach to monitor protein *S*-persulfidation levels in live cells by tracking H_2_S_2_/H_2_S_*n*_ dynamics. In H9c2 cardiomyocytes, treatment with donor JPT-1 effectively enhancing H_2_S_2_/H_2_S_*n*_ levels. This represents the cellular-level demonstration of thiol-dependent modulation of *S*-persulfidation, with implications for redox regulation and thiol-based therapeutic strategies.

Overall, the Cyne probe series provides a powerful platform for dissecting H_2_S_2_/H_2_S_*n*_ chemistry *in vitro* an *in vivo*, guiding the design of next-generation persulfide donors, and enabling mechanistic insights into thiol-specific protein modifications.

Hydrogen persulfide/polysulfides have emerged as potential gasotransmitters with important physiological functions and are also implicated in various pathological processes. Studies have shown that H_2_S_2_/H_2_S_*n*_ levels undergo significant alterations in multiple disease models. For example, they are markedly elevated in inflammatory conditions and type 2 diabetes, whereas they exhibit a decreasing trend in liver injury models. Moreover, H_2_S_2_/H_2_S_*n*_ has been demonstrated to play a critical role in regulating ferroptosis. We believe that the development of highly specific and sensitive detection tools, such as the Cyne series probes, will not only offer reliable means for monitoring disease progression but also help uncover the underlying molecular mechanisms in these disease contexts. Therefore, future studies will focus on mapping the spatiotemporal dynamics of H_2_S_2_/H_2_S_*n*_ in disease models to better understand their signaling roles and therapeutic potential.

## Author contributions

Xidan Tong, Xiaowei Xu, and Jiaxuan Chen contributed equally to this work. Weiwei Guo, Zhen Li and Yueqin Zheng supervised the study. Xidan Tong and Yueqin Zheng conceived the initial idea and designed the research. Xidan Tong and Xiaowei Xu designed and synthesized the compounds with assistance from Jinkang Feng and Yixing Li. Xidan Tong and Xiaowei Xu designed and performed the biological experiments with support from Yangfei Shi and Jiaxuan Chen. Xidan Tong and Jiaxuan Chen drafted the manuscript. All authors reviewed, edited, and approved the final version of the manuscript.

## Conflicts of interest

The authors declare no competing financial interest.

## Appendix A Supporting information

The following is the Supporting information to this article:
