# Supplementary material for: Ultrasensitive bioorthogonal probes for selective discrimination of trace H2S2/H2Sn in sulfide-competitive contexts
Source: Acta Pharm Sin B. 2026 Feb 4;16(7):4539–51. doi: 10.1016/j.apsb.2026.01.044 (PMC13366304; doi:10.1016/j.apsb.2026.01.044)
Supplement: Multimedia component 1 [file mmc1.pdf]

Supporting Information for

Original article

Ultrasensitive bioorthogonal probes for selective discrimination of Trace H_2S_2/H_2S_n in sulfide-competitive contexts

Xidan Tong[a,†], Xiaowei Xu[a,†], Jiaxuan Chen[a,†], Jinkang Feng[a], Yixing Li[a], Yangfei Shi[a], Zhen Li[c], Weiwei Guo[b], Yueqin Zheng[a,*]

[a]*State Key Laboratory of Natural Medicines and Jiangsu Key Laboratory of Drug Discovery for Metabolic Diseases, Center of Drug Discovery, China Pharmaceutical University, Nanjing 211198, China*

[b]*Department of Chemistry, China Pharmaceutical University, Nanjing 211198, China*

[c]*State Key Laboratory of Natural Medicines, School of Traditional Chinese Pharmacy, China Pharmaceutical University, Nanjing 211198, China*

[†]These authors made equal contributions to this work.

Received 13 August 2025; received in revised form 5 December 2025; accepted 7 December 2025

[*]Corresponding author.

E-mail addresses: yzheng@cpu.edu.cn (Yueqin Zheng).

1. General materials and methods

All chemicals purchased from commercial suppliers and were used as received unless otherwise stated. All solvents were reagent grade and, when necessary, were purified and dried by standard methods. Reactions were monitored by thin-layer chromatography (TLC) on 0.25 mm silica gel plates (GF254) and visualized under ultraviolet (UV) light. ^{1}H NMR and ^{13}C NMR spectra were recorded with Bruker AV-300, Bruker AV-400 or Bruker AV-600 spectrometers at 300 K. Tetramethylsilane (TMS) was used as an internal standard and chemical shifts were reported in parts per million (ppm). All coupling constants (*J*) are in hertz (Hz), and the signals are designated as follows: s, singlet; d, doublet; t, triplet; q, quartet; m, multiplet; br s, broad singlet. High-resolution mass spectra (HRMS) were recorded on Agilent Q-TOF 6520 mass spectrometer with electron spray ionization (ESI) as the ion source. The purity of all compounds in this experiment was identified by HPLC and ^{1}H, ^{13}C NMR, and the purity was ≥ 95%.

Dulbecco's modified Eagle's medium (DMEM), Trypsin–EDTA (0.05%), penicillin–streptomycin (10,000 U/mL), and fetal bovine serum (FBS) were purchased from Gibco. The Cell Counting Kit-8 (CCK-8) was purchased from Beyotime Biotechnology Co., Ltd. 4T1 cells, HEK293T cells, and H9c2 cardiomyocytes were obtained from American Type Culture Collection (ATCC). BALB/c mice (aged 6–7 weeks, female, SPF grade, 18–22 g) were purchased from Jiangsu Qinglongshan Biotechnology Co., Ltd.

2. Chemical synthesis

2.1. Synthesis of the probes ***Cyne-1–5***

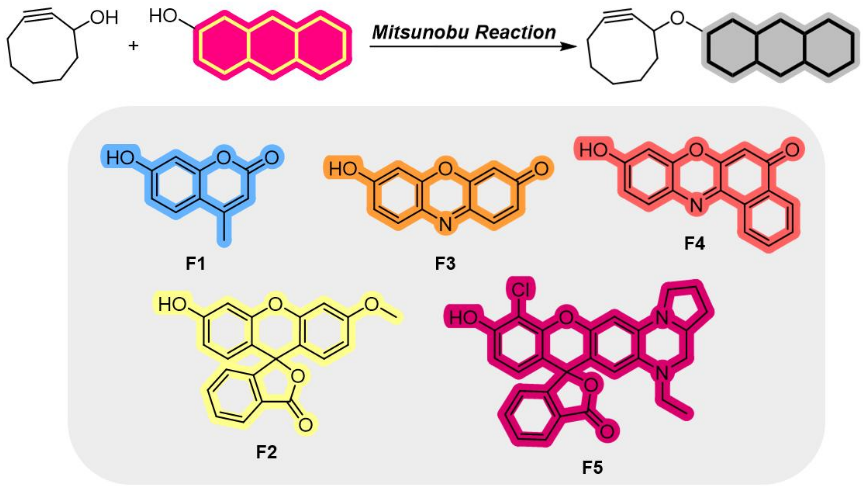

General Scheme:

Probes **Cyne1~5** were synthesized by one-step Mitsunobu reaction of cyclooct-2-yn-1-ol (**Cyne-OH**) and different fluorophores. Different reaction conditions (such as temperature, solvent and reaction time) were adopted according to the properties of different fluorophores (solubility, pKa, steric hindrance, etc.). Please see below for specific reaction conditions.

Cyclooct-2-yn-1-ol (**Cyne-OH**) and the fluorophores **F2**, **F4**, and **F5** were synthesized according to reported protocols[1-4]. The spectrum data of the product is consistent with the literature report.

*2.1.1 Synthesis of probe **Cyne-1***

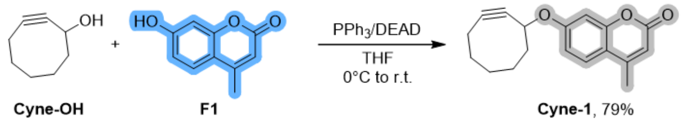

Under argon atmosphere, 7-hydroxy-4-methyl-2*H*-chromen-2-one (**F1**, 141.3 mg, 802 µmol, 1.2 equiv), triphenylphosphine (210.4 mg, 802 µmol, 1.2 equiv) and diethyl azodicarboxylate (DEAD, 140 µL, 802 µmol, 1.2 equiv) were sequentially added to a solution of cyclooct-2-yn-1-ol (**Cyne-OH**, 83.0 mg, 668 µmol, 1.0 equiv) in anhydrous tetrahydrofuran (2.0 mL) at 0 °C. The reaction mixture was stirred at 0°C for 30 min, and then warmed to room temperature for 2 h. After competition, the solution was concentrated under reduced pressure, and purified by column chromatography on silica gel (eluent: pure DCM) to provide **Cyne-1** (149 mg, 79%) as a white solid.

TLC (pure DCM), R*f*: 0.68 (UV visualization and $KMnO_4$)

^{1}H NMR (300 MHz, $CDCl_3$) δ 7.49 – 7.41 (m, 1H), 6.89 – 6.81 (m, 2H), 6.09 (s, 1H), 4.84 – 4.76 (m, 1H), 2.35 (s, 3H), 2.22 (dq, J = 20.2, 5.7 Hz, 4H), 1.88 (h, J = 5.0, 4.4 Hz, 3H), 1.73 – 1.56 (m, 3H).

^{13}C NMR (75 MHz, $CDCl_3$) δ 161.42, 160.79, 152.68, 125.54, 113.91, 113.02, 112.13, 102.85, 102.71, 90.68, 70.61, 42.18, 34.19, 29.71, 26.15, 20.77, 18.78.

HRMS(ESI)(*m/z*): calc'd for $C_{18}H_{19}O_3$ $[M+H]^+$: 283.1328, found: 283.1317

*2.1.2 Synthesis of probe **Cyne-2***

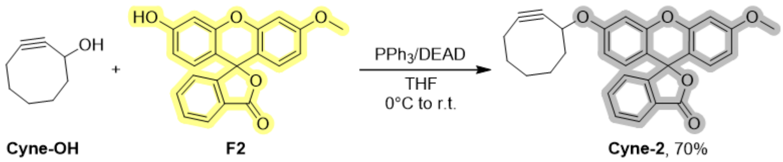

Under argon atmosphere, 3′-hydroxy-6′-methoxy-*3H*-spiro[isobenzofuran-1,9′-xanthen]-3-one (**F2**, 409 mg, 1182 µmol, 1.5 equiv), triphenylphosphine (248 mg, 946 µmol, 1.2 equiv) and diethyl azodicarboxylate (DEAD, 151 µL, 946 µmol, 1.2 equiv) were sequentially added to a solution of cyclooct-2-yn-1-ol (**Cyne-OH**, 98 mg, 788 µmol, 1.0 equiv) in anhydrous tetrahydrofuran (10.0 mL) at 0 °C. The reaction mixture was stirred at 0 °C for 30 min, and then warmed to room temperature for 1 h. After competition, the solution was concentrated under reduced pressure, and purified by column chromatography on silica gel (eluent: pure DCM) to provide **Cyne-2** (250 mg, 70%) as a white solid.

TLC (pure DCM), R*f*: 0.80 (UV visualization and $KMnO_4$)

^{1}H NMR (300 MHz, $CDCl_3$) δ 8.01 (d, J = 7.1 Hz, 1H), 7.69 – 7.57 (m, 2H), 7.14 (d, J = 7.2 Hz, 1H), 6.83 (d, J = 2.4 Hz, 1H), 6.77 (d, J = 2.5 Hz, 1H), 6.67 (dtt, J = 7.8, 5.4, 2.4 Hz, 3H), 6.62 – 6.58 (m, 1H), 4.86 – 4.76 (m, 1H), 3.83 (s, 3H), 2.34 – 2.19 (m, 4H), 1.92 (tt, J = 10.5, 6.1 Hz, 3H), 1.77 – 1.62 (m, 3H).

^{13}C NMR (75 MHz, $CDCl_3$) δ 169.55, 161.33, 159.40, 153.31, 152.32, 134.99, 129.68, 129.10, 125.02, 124.03, 113.04, 112.13, 111.63, 111.27, 102.87, 102.24, 102.07, 100.86, 91.12, 83.33, 77.30, 70.29, 55.62, 42.22, 34.19, 29.73, 26.15, 20.80.

HRMS(ESI)(*m/z*): calc'd for $C_{29}H_{25}O_5$ $[M+H]^+$: 453.1697, found: 453.1699

2.1.3 Synthesis of probe ***Cyne-3***

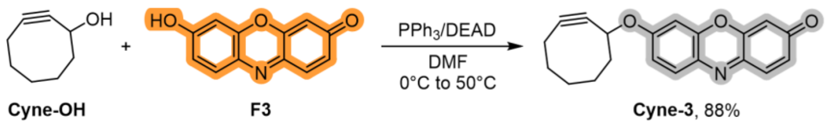

Under argon atmosphere, 7-hydroxy-*3H*-phenoxazin-3-one (Resorufin, **F3**, 110 mg, 516 µmol, 1.2 equiv), triphenylphosphine (135 mg, 516 µmol, 1.2 equiv) and diethyl azodicarboxylate (DEAD, 99 µL, 516 µmol, 1.2 equiv) were sequentially added to a solution of cyclooct-2-yn-1-ol (**Cyne-OH**, 53 mg, 427 µmol, 1.0 equiv) in anhydrous DMF (2.0 mL) at 0°C. The reaction mixture was stirred

at 0 °C for 30 min, and then heated to 50 °C for 3 h. After competition, the solution was concentrated under reduced pressure, and purified by column chromatography on silica gel (eluent: 3% MeOH in DCM) to provide **Cyne-3** (120 mg, 88%) as a light orange solid. ***Note***: The concentration of the Mitsunobu reaction is critical to the yield of the product. Since resorufin has a poor solubility in THF, we strongly recommend using DMF as the reaction solvent for the synthesis of probe **Cyne-3**. (Yield of **Cyne-3**: 88% in DMF, 16% in THF, 15% in ACN)

TLC (5% MeOH in DCM), R*f*: 0.65 (UV visualization and $KMnO_4$)

^{1}H NMR (600 MHz, $CDCl_3$) δ 7.69 (d, J = 8.9 Hz, 1H), 7.41 (d, J = 9.8 Hz, 1H), 6.95 (dd, J = 8.9, 2.7 Hz, 1H), 6.88 (d, J = 2.6 Hz, 1H), 6.83 (dd, J = 9.8, 2.1 Hz, 1H), 6.32 (d, J = 2.1 Hz, 1H), 4.85 (td, J = 5.6, 2.7 Hz, 1H), 2.31 – 2.19 (m, 4H), 1.94 (qt, J = 8.2, 3.0 Hz, 4H), 1.79 – 1.74 (m, 1H), 1.67 – 1.64 (m, 1H).

^{13}C NMR (151 MHz, $CDCl_3$) δ 186.31, 149.88, 145.66, 134.69, 134.23, 131.42, 128.54, 114.67, 106.72, 103.29, 101.63, 90.26, 71.09, 42.12, 34.10, 29.60, 26.06, 20.69.

HRMS(ESI)(*m/z*): calc'd for $C_{20}H_{18}NO_3$ $[M+H]^+$: 320.1281, found: 320.1279

2.1.4 Synthesis of probe ***Cyne-4***

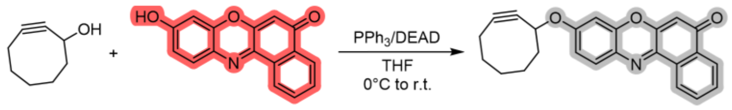

Under argon atmosphere, 9-hydroxy-5*H*-benzo[a]phenoxazin-5-one (NileRed, **F4**, 50 mg, 186 µmol, 1.1 equiv), triphenylphosphine (69 mg, 254 µmol, 1.5 equiv) and diethyl azodicarboxylate (DEAD, 49 µL, 254 µmol, 1.5 equiv) were sequentially added to a solution of cyclooct-2-yn-1-ol (**Cyne-OH**, 21 mg, 169 µmol, 1.0 equiv) in anhydrous tetrahydrofuran (2.5 mL) at 0 °C. The reaction mixture was stirred at 0 °C for 30 min, and then warmed to room temperature for 5 h. After competition, the solution was concentrated under reduced pressure, and purified by column chromatography on silica gel (eluent: 20% hexane in EtOAc) to provide **Cyne-4** (44.5 mg, 62%) as a dark red solid.

TLC (20% hexane in EtOAc), R*f*: 0.70 (UV visualization and $KMnO_4$)

^{1}H NMR (600 MHz, $CDCl_3$) δ 8.61 (d, J = 7.7 Hz, 1H), 8.25 (dd, J = 7.7, 1.6 Hz, 1H), 7.72 – 7.65

(m, 3H), 6.89 (dd, J = 8.7, 2.7 Hz, 1H), 6.82 (d, J = 2.7 Hz, 1H), 6.37 (s, 1H), 4.83 – 4.79 (m, 1H), 2.31 – 2.20 (m, 4H), 1.92 (tdd, J = 12.3, 6.1, 2.1 Hz, 3H), 1.79 – 1.74 (m, 1H), 1.70 – 1.63 (m, 2H).

^{13}C NMR (151 MHz, $CDCl_3$) δ 183.74, 160.59, 144.28, 132.01, 131.74, 131.48, 131.10, 130.70, 125.79, 124.31, 113.89, 106.91, 102.93, 101.67, 90.58, 70.88, 42.16, 34.13, 29.64, 26.10, 20.71.

HRMS(ESI)(*m/z*): calc'd for $C_{24}H_{20}NO_3$ $[M+H]^+$: 370.1438, found: 370.1438

2.1.5 Synthesis of probe ***Cyne-5***

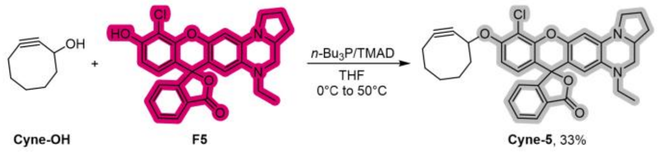

Under argon atmosphere, 11-chloro-5-ethyl-10-hydroxy-1,2,3,3a,4,5-hexahydro-3'*H*-spiro[chromeno[3,2-*g*]pyrrolo[1,2-*a*]quinoxaline-7,1′-isobenzofuran]-3′-one (**F5**, 50 mg, 105 μmol, 1.5 equiv), tributylphosphine (69 mg, 140 μmol, 2.0 equiv) and *N,N,N′,N′*-tetramethylazodicarboxamide (TMAD, 24.1 mg, 140 μmol, 2.0 equiv) were sequentially added to a solution of cyclooct-2-yn-1-ol (**Cyne-OH**, 8.8 mg, 70 μmol, 1.0 equiv) in anhydrous tetrahydrofuran (1.0 mL) at 0 °C. The reaction mixture was stirred at 0 °C for 30 min, and then heated to 50 °C for 6 h. After competition, the solution was concentrated under reduced pressure, and purified by column chromatography on silica gel (eluent: 5% MeOH in DCM) to provide **Cyne-5** (13.6 mg, 33%) as a dark purple solid. ***Note***: When using DEAD/PPh_3, the substrate conversion rate is only 2% in 5 h (identified by LCMS). In this reaction, we recommend using the more active TMAD as the oxidant.

TLC (5% MeOH in DCM), R*f*: 0.28 (UV visualization and $KMnO_4$)

^{1}H NMR (600 MHz, MeOD) δ 8.30 (t, *J* = 7.5 Hz, 1H), 7.84 – 7.72 (m, 2H), 7.43 – 7.36 (m, 1H), 7.08 (t, *J* = 10.4 Hz, 1H), 6.98 (s, 1H), 6.02 (d, *J* = 9.9 Hz, 1H), 3.88 (s, 2H), 3.66 (s, 1H), 2.97 (t, *J* = 11.3 Hz, 1H), δ 2.21 (dt, *J* = 15.1, 7.3 Hz, 2H), 2.15 – 2.11 (m, 1H), 2.11 – 2.04 (m, 2H), 1.94 – 1.87 (m, 2H), 1.83 – 1.81 (m, 1H), 1.65 (ddd, *J* = 26.6, 16.9, 9.9 Hz, 4H), 1.43 (dt, *J* = 16.9, 8.8 Hz, 2H), 0.98 (s, 3H).

^{13}C NMR (151 MHz, MeOD) δ 163.57, 163.32, 162.55, 157.15, 155.59, 151.38, 150.41, 136.22, 135.58, 135.49, 133.43, 133.19, 131.40, 131.32, 131.20, 128.44, 119.07, 118.98, 117.64, 117.22,

117.02, 109.00, 102.97, 96.56, 95.27, 90.54, 71.55, 65.10, 59.83, 50.51, 50.27, 49.83, 49.41, 49.27, 49.13, 48.99, 48.84, 48.70, 48.56, 46.78, 46.28, 42.29, 35.54, 31.01, 27.26, 23.69, 21.19, 9.60.

HRMS(ESI)(*m/z*): calc'd for $C_{24}H_{20}NO_3$ $[M+H]^+$: 581.2202, found: 581.2215

*2.2. Synthesis of the compounds **Cene-1**, **Lyne-1** and **CM-CT***

Compounds **CM-CT** and **Lyne-1** were synthesized according to reported protocols[5-6]. The spectrum data of the product is consistent with the literature report.

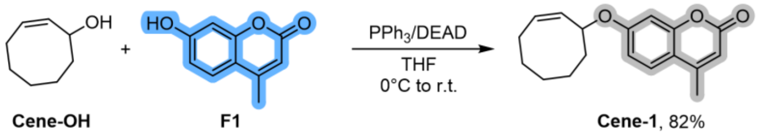

Under argon atmosphere, 7-hydroxy-4-methyl-2*H*-chromen-2-one (**F1**, 141.3 mg, 802 µmol, 1.2 equiv), triphenylphosphine (210.4 mg, 802 µmol, 1.2 equiv) and diethyl azodicarboxylate (DEAD, 140 µL, 802 µmol, 1.2 equiv) were sequentially added to a solution of (*Z*)-cyclooct-2-en-1-ol (**Cene-OH**, 84.3 mg, 668 µmol, 1.0 equiv) in anhydrous tetrahydrofuran (2.0 mL) at 0°C. The reaction mixture was stirred at 0°C for 30 min, and then warmed to room temperature for 2 h. After competition, the solution was concentrated under reduced pressure, and purified by column chromatography on silica gel (eluent: pure DCM) to provide **Cene-1** (155 mg, 82%) as a white solid.

TLC (pure DCM), R*f*: 0.65 (UV visualization and $KMnO_4$)

^{1}H NMR (300 MHz, $CDCl_3$) δ 7.46 (d, *J* = 8.7 Hz, 1H), 6.88 – 6.71 (m, 2H), 6.16 – 6.04 (m, 1H), 5.88 – 5.71 (m, 1H), 5.45 (dd, *J* = 10.8, 7.1 Hz, 1H), 5.21 – 5.02 (m, 1H), 2.42 – 2.35 (m, 3H), 2.33 – 2.19 (m, 2H), 2.10 (td, *J* = 8.4, 4.2 Hz, 1H), 1.83 – 1.43 (m, 8H).

^{13}C NMR (75 MHz, $CDCl_3$) δ 161.37, 155.10, 152.75, 131.77, 131.05, 125.36, 113.60, 113.23, 111.57, 102.34, 76.02, 35.61, 28.96, 26.82, 26.12, 23.30, 18.68.

HRMS(ESI)(*m/z*): calc'd for $C_{18}H_{20}O_3$ $[M+H]^+$: 285.1485, found: 285.1489

*2.3. Synthesis of persulfide donors **Pre-PenSSH**, **Pre-tBuSSH** and **Pre-EtSSH***

Two thioisothiourea persulfide donors **Pre-PenSSH** and **Pre-tBuSSH** were synthesized according to the literature reported by *Toscano* group[7]. Trimethyl lock-type persulfide donor **Pre-EtSSH** was

synthesized according to our previous work[5]. The spectrum data of the product is consistent with the literature report.

2.4. Synthesis of thiocarboxylic acid-based H_2S donors ***ZYSA02*** *and* ***ZYSH09***

The thiocarboxylic acid-based H_2S donors **ZYSA02** and **ZYSH09** were synthesized according to our previous work. The spectrum data of the product is consistent with the literature report.

2.5. Synthesis of arylsulfonothioate donor ***JPT-1***

The arylsulfonothioate donor **JPT-1** was synthesized according to the literature reported by *Toscano* group[8]. The spectrum data of the product is consistent with the literature report.

3. Fluorescence photophysical property measurements

All fluorescence measurements were carried out at room temperature (25 °C) on an Agilent Cary Eclipse fluorescence spectrophotometer. The testing solutions of the probe were excited at the maximum excitation wavelength of the corresponding fluorophore (see Table 1), and the excitation and emission slit widths were set to 5/5 nm. The emission spectrum was scanned in the appropriate range (see Figure S4) at 1200 nm/min and the photomultiplier voltage was set at 400 V. The probe **Cyne-1~5** was dissolved in DMSO to make a 1 mM stock solution, which was diluted to the required concentration of testing solution for measurement. Aliquots of analyte solutions were slowly added to probe testing solution (3 mL) with vigorous stirring at room temperature in the dark. ***Note***: The volume changes after addition of analyte solutions were less than 1%. For time-dependent measurements, the fluorescence intensity of the test solution was recorded at one-minute intervals over a period of 30 minutes. For concentration-dependent measurement, the fluorescence intensities of the testing solutions were recorded after 30 min.

4. The fluorescence quantum yield (Φ) measurement

The fluorescence quantum yield was determined using rhodamine 6G in ethanol solution ($\Phi_f = 0.94$) or eminaphthofluorescein-10 in pH 11 Glycine-NaOH buffer ($\Phi_f = 0.35$) as a reference standard[9-10], and calculated according to the following equation:

$$\Phi_{f(X)} = \Phi_{f(S)} \, (A_S F_X / A_X F_S) \, (n_X / n_S)^2$$

Where Φ_f represents the fluorescence quantum yield, **A** denotes the absorbance at the excitation wavelength, **F** corresponds to the integrated area under the corrected emission curve, **n** refers to the refractive index of the solvent employed, while the subscripts **S** and **X** designate the standard and test samples, respectively.

5. HPLC experimental method

High-performance liquid chromatography (HPLC) analyses were performed on an Agilent 1290 system equipped with a diode array detector (DAD). Separation was achieved on a C18 reverse-phase column (150 mm × 4.6 mm, 5 μm particle size) maintained at 35 °C. The mobile phase comprised solvent A (water containing 0.1% trifluoroacetic acid, TFA) and solvent B (acetonitrile with 0.1% TFA). A gradient elution program was applied as follows: initial 10% B, linearly increased to 90% B over 8 min, held for 2 min, and then returned to the initial conditions. The flow rate was 1.0 mL/min, and detection was performed at 254 nm. The injection volume was 20 μL, and the total analysis time per sample was approximately 15 min. Quantification was based on calibration curves constructed from standard solutions of the target compound.

6. Preparation of analyte solutions

The preparation of analyte solutions was processed according to the methods reported in the literature[11-14], with some appropriate modifications (*vide infra*).

6.1. ROS species

H_2O_2: H_2O_2 solution (10 mmol/L) was added directly.

t-BuOOH: tert-Butyl hydroperoxide solution (10 mmol/L) was added into the testing solutions directly.

$^1O_2$: Singlet oxygen was generated from 3,3'-(naphthalene-1,4-diyl)dipropionic acid (10 mmol/L).

O_2^-: KO2 solution (10 mmol/L) was added directly.

HOCl: NaOCl solution (10 mmol/L) was added directly. ***<u>Note:</u>*** The concentration of the NaClO solution is calibrated via iodometry prior to each test.

•OH: Hydroxyl radical was generated by Fenton reaction or TCBQ/H_2O_2. To generate •OH, ferrous chloride or TCBQ solution was added to a solution of H_2O_2 (10 equiv). The concentration of •OH

was equal to the Fe(II) concentration (5 mmol/L) or TCBQ concentration (5 mmol/L).

$ONOO^-$: A mixture of sodium nitrite (0.6 mol/L) and hydrogen peroxide (0.7 mol/L) was acidified with hydrochloric acid (0.6 mol/L) and sodium hydroxide (1.5 mol/L) was added within 1–2 s to make the solution alkaline. Excess hydrogen peroxide was removed by passing the solution through a short column of manganese dioxide. The resulting solution was divided into small aliquots and stored below –18°C. These aliquots were thawed immediately before use and the concentration of peroxynitrite was determined by measuring the absorbance of the solution at 302 nm (the extinction coefficient of a peroxynitrite solution in 0.1 mol/L NaOH at 302 nm is 1670 M^{-1} cm^{-1}).

6.2. RNS species

NO: Nitric oxide was generated from SNP (sodium nitroferricyanide (III) dihydrate) (5 mmol/L).

NO_2^-: Freshly prepared sodium nitrite solution (5 mmol/L) was added directly.

NO_3^-: Freshly prepared sodium nitrate solution (5 mmol/L) was added directly.

6.3 RSS species

Cys: Cysteine solution (10 mmol/L) was added directly.

Hcy: Homocysteine solution (10 mmol/L) was added directly.

Met: Methionine solution (10 mmol/L) was added directly.

GSH: Reduced glutathione solution (10 mmol/L) was added directly.

GSSG: Oxidized glutathione solution (10 mmol/L) was added directly.

CH_3SSSCH_3: Dimethyl trisulfide solution (10 mmol/L in DMSO) was added directly.

S_8: The stock solution of S_8 (200 mmol/L) was prepared in DCM, and then used EtOH to dilute this solution, to get a 10 mmol/L stock solution of S_8 in EtOH.

Na_2S: $Na_2S \cdot 9H_2O$ solution (10 mmol/L) was added directly.

Na_2S_2: Na_2S_2 solution (100 mmol/L) was added directly.

Na_2S_4: Na_2S_4 solution (100 mmol/L) was added directly. ***Note***: Na_2S_n is relatively unstable at low concentrations and should be stored at higher concentrations. The stock solution must be prepared freshly for each test.

6.4. Other species

Esterase: Freshly prepared porcine liver esterase (PLE, 30 unit/mL) was added directly

Other amino acids (see Figure S7): The corresponding amino acids solution (10 mmol/L) was added directly.

Other inorganic salts/metal ions (see Figure S7): The corresponding inorganic salts/metal ions solution (10 mmol/L) was added directly.

Vitamin C: Freshly prepared sodium ascorbate (10 mmol/L) was added directly.

7. Cell experiment

4T1 cells (obtained from American Type Culture Collection, ATCC) were cultured in RPMI 1640 medium with 1% penicillin–streptomycin and 10% fetal bovine serum (FBS) at 37 °C in a humidified environment composed of 95% air and 5% CO_2.

HEK293T cells and H9c2 cardiomyocytes (obtained from American Type Culture Collection, ATCC) were cultured in DMEM (Dulbecco's modified Eagle medium; high glucose) with 1% penicillin-streptomycin and 10% fetal bovine serum (FBS) at 37 °C in a humidified environment composed of 95% air and 5% CO_2.

7.1 Cytotoxicity assay

4T1 cells in the logarithmic growth phase were seeded on 96-well plates at a density of 5×10^3 cells per well. Following a 24-hour incubation at 37 °C in a 5% CO_2 atmosphere, the cells were treated to the tested compounds at different concentrations for an additional 24 h. Subsequently, the cells were washed twice with PBS and treated with 10% CCK-8 for 1 hour at 37 °C. The absorbance at 450 nm was then recorded using a microplate reader. Cell viability was assessed by calculating the ratio of experimental wells (after subtracting background) to control wells (also after subtracting background). The results were summarized in Figures S12.

7.2 Cellular imaging

4T1 cells were grown on glass-bottom culture dishes in RPMI 1640 medium supplemented with 1% penicillin-streptomycin and 10% FBS at 37°C in a humidified environment composed of 95% air and 5% CO_2. Before use, the adherent cells were washed two times with FBS-free RPMI 1640 medium or PBS buffer. Subsequently, cells were subjected to different treatments according to the

experimental requirements. For ease of reading, detailed operational procedures have been included in the manuscript and corresponding figure captions. To avoid redundancy, it will not be repeated here. Following the removal of the culture solution, cellular imaging was conducted using an Olympus FV-3000 confocal laser scanning microscope (CLSM).

H9c2 cardiomyocytes were grown on glass-bottom culture dishes in DMEM supplemented with 1% penicillin-streptomycin and 10% FBS at 37 °C in a humidified environment composed of 95% air and 5% CO_2. Before use, the adherent cells were washed two times with FBS-free DMEM or PBS buffer. Subsequently, cells were subjected to different treatments according to the experimental requirements. For ease of reading, detailed operational procedures have been included in the manuscript and corresponding figure captions. To avoid redundancy, it will not be repeated here. Following the removal of the culture solution, cellular imaging was conducted using an Olympus FV-3000 confocal laser scanning microscope (CLSM).

7.3 Flow cytometry analysis

4T1 cells were plated in six-well plates at a density of 5×10^5 cells/mL (2 mL/well in triplicates) 24 hours before assay. The medium used was RPMI 1640 medium supplemented with 10% FBS and 1% penicillin/streptomycin. For fluorescence measurement, all reagents and probe solutions were freshly dissolved in phenolsulfonphthalein indicator-free indicator RPMI 1640 medium. Prior to the experiment, discard the culture medium and wash the cells twice with PBS buffer. Subsequently, cells were subjected to different treatments according to the experimental requirements. For ease of reading, detailed operational procedures have been included in the manuscript and corresponding figure captions. To avoid redundancy, it will not be repeated here. Finally, after centrifugation and washing, the cells were resuspended in PBS and analyzed for fluorescence intensity using a flow cytometer (Agilent Technologies NovoCyte). E_x wavelength: 488 nm; E_m channel: 530/30 nm. Data are presented, without subtracting background fluorescence, as mean ± s.d. for fluorescence measurement with replicates (n = 5) in at least three independent experiments.

HEK293T cells were plated in six-well plates at a density of 5×10^5 cells/mL (2 mL/well in triplicates) 24 hours before assay. The medium used was DMEM supplemented with 10% FBS and 1% penicillin/streptomycin. For fluorescence measurement, all reagents and probe solutions were freshly dissolved in phenolsulfonphthalein indicator-free indicator DMEM. Prior to the experiment,

discard the culture medium and wash the cells twice with PBS buffer. Subsequently, cells were subjected to different treatments according to the experimental requirements. For ease of reading, detailed operational procedures have been included in the manuscript and corresponding figure captions. To avoid redundancy, it will not be repeated here. Finally, after centrifugation and washing, the cells were resuspended in PBS and analyzed for fluorescence intensity using a flow cytometer (Agilent Technologies NovoCyte®). E_x wavelength: 488 nm; E_m channel: 530/30 nm. Data are presented, without subtracting background fluorescence, as mean ± s.d. for fluorescence measurement with replicates (n = 5) in at least three independent experiments.

8. *In vivo* NIR fluorescence imaging

All animal experiment protocols were approved by the Animal Care and Use Committee of China Pharmaceutical University and comply with all relevant ethical regulations.

For near-infrared imaging *in vivo*, images for healthy BALB/c mice (aged 6–7 weeks, female, SPF grade, 18–22 g) anesthetized with isoflurane were recorded at various times after the probe **Cyne-5** injection using PerkinElmer IVIS® Lumina LT Series III *in vivo* imaging system. Excitation filter: 570 nm; Emission filter: 660 nm.

9. Supplementary Figures and Tables

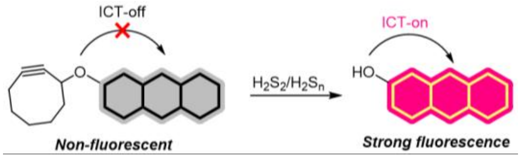

Figure S1. The design of the ether-based probe and the principle of fluorescence quenching.

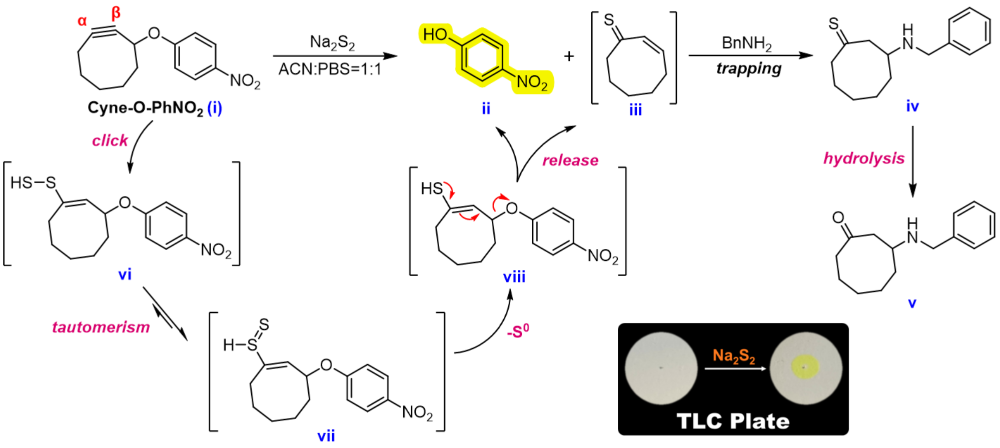

Figure S2. Proposed detection mechanism of **Cyne** probes.

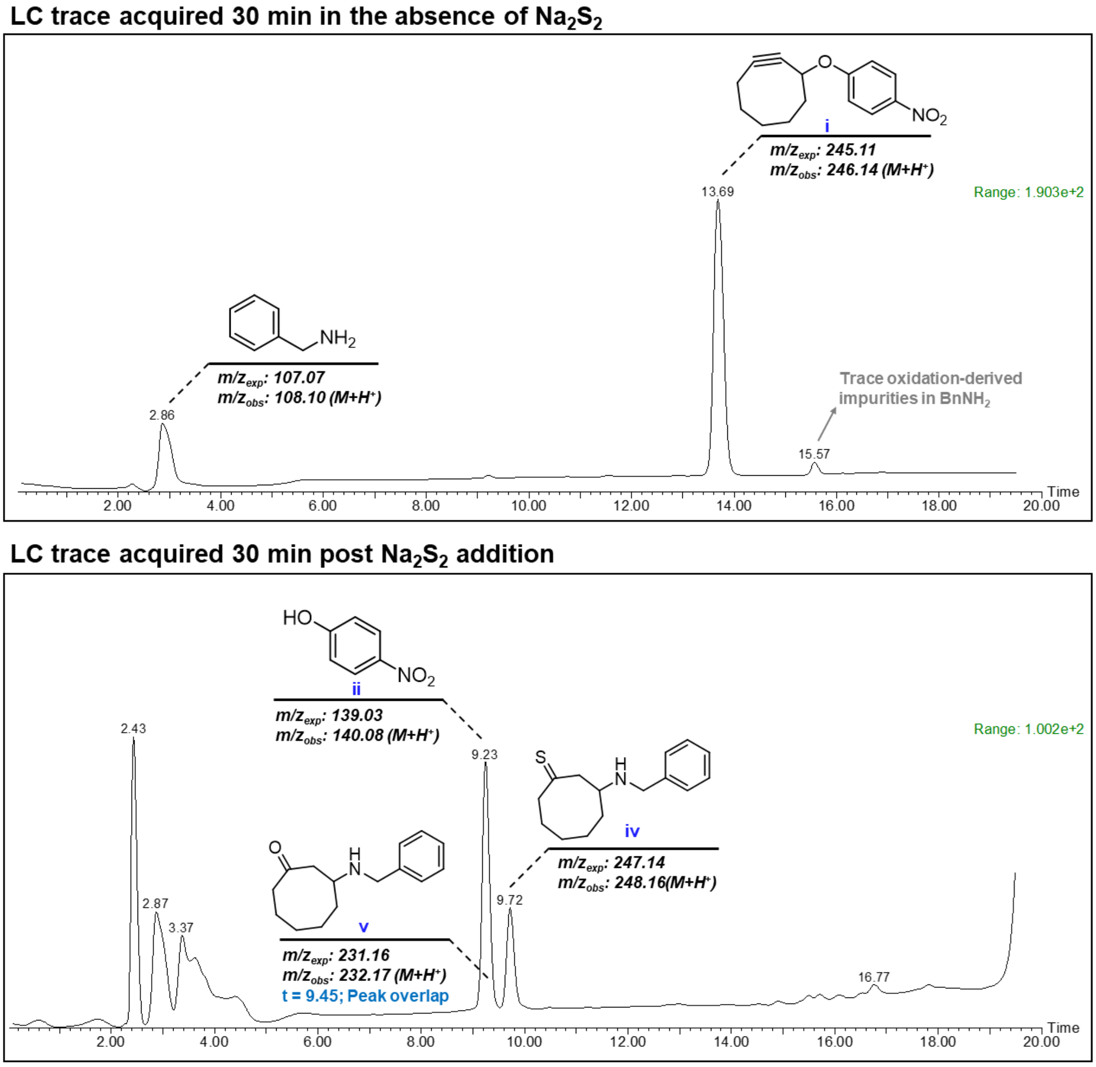

MS trace of Cyne-O-PhNO$_2$ (i)

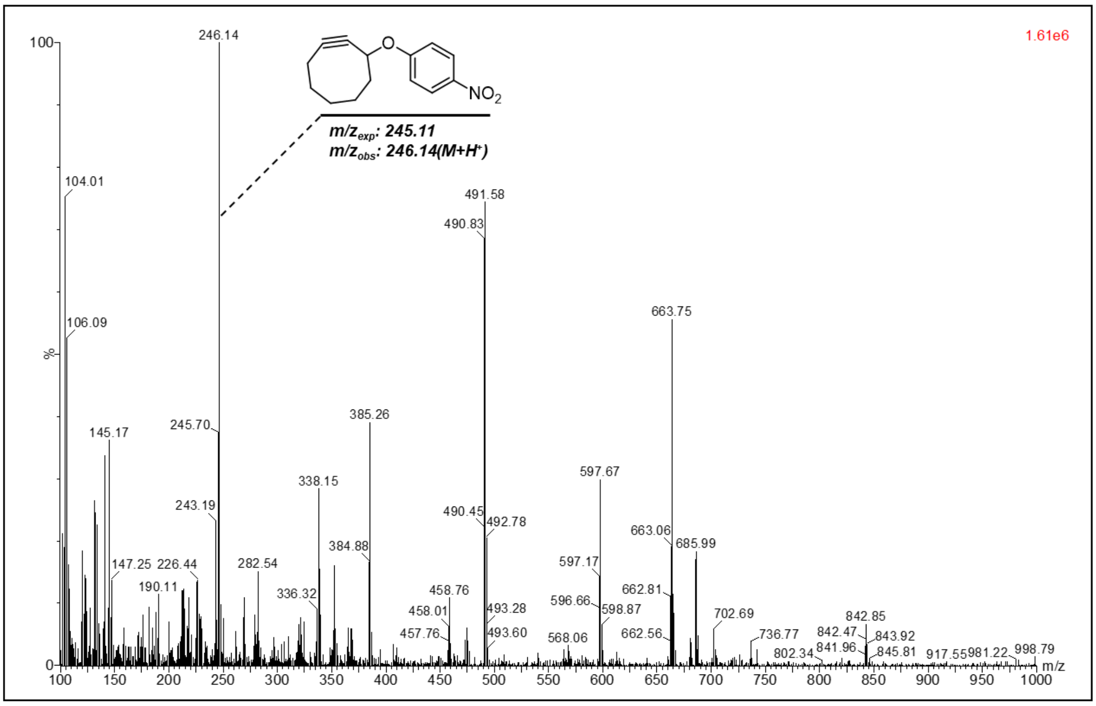

MS trace of trapped product thioketone iv

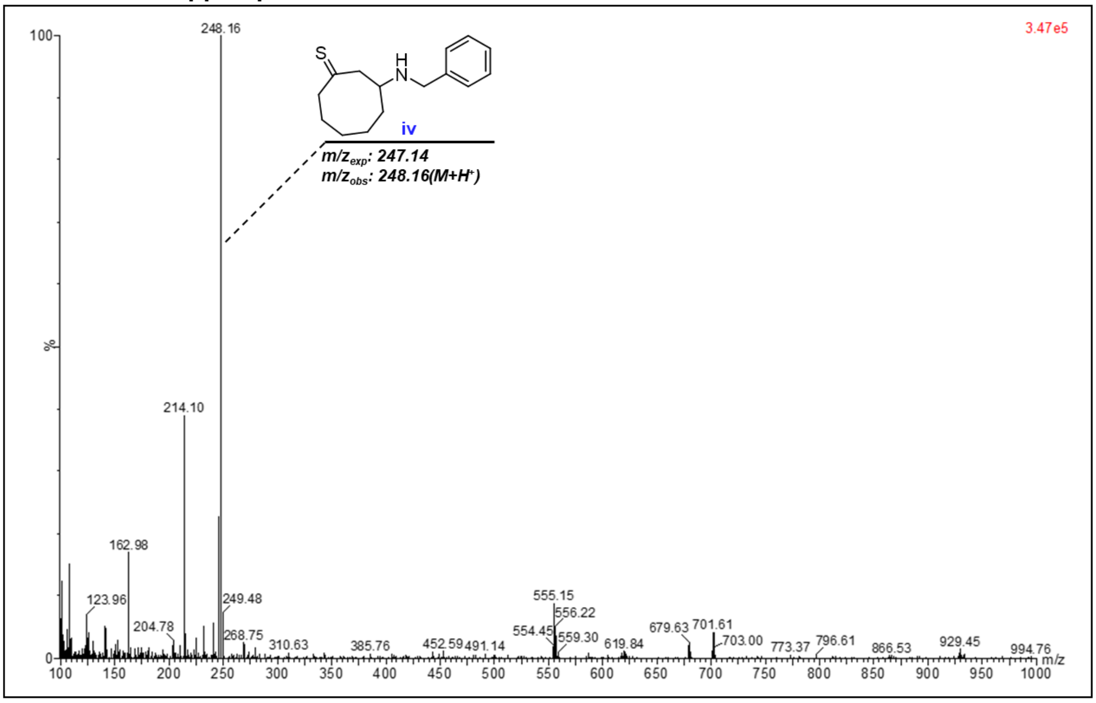

MS trace of trapped product ketone v

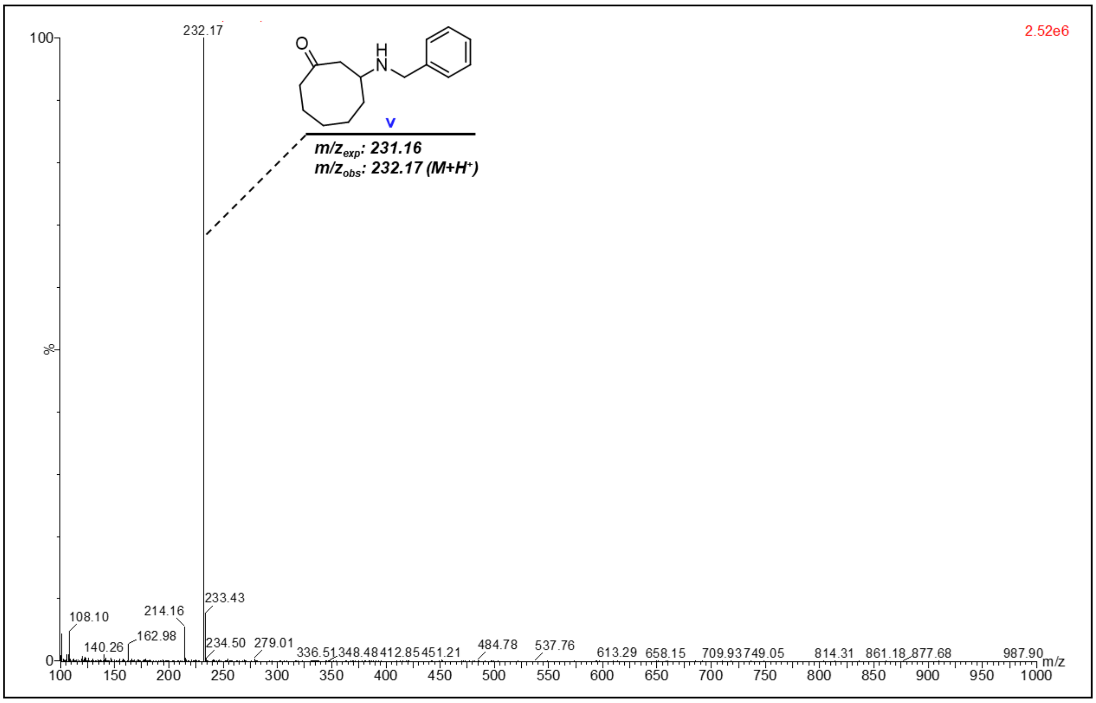

Figure S3. Reaction of probe **Cyne-O-PhNO₂** (1 mM) with Na_2S_2 (10 mM) in ACN:PBS (1:1, pH 7.4, r.t.) in the presence of benzylamine ($BnNH_2$, 10 mM).

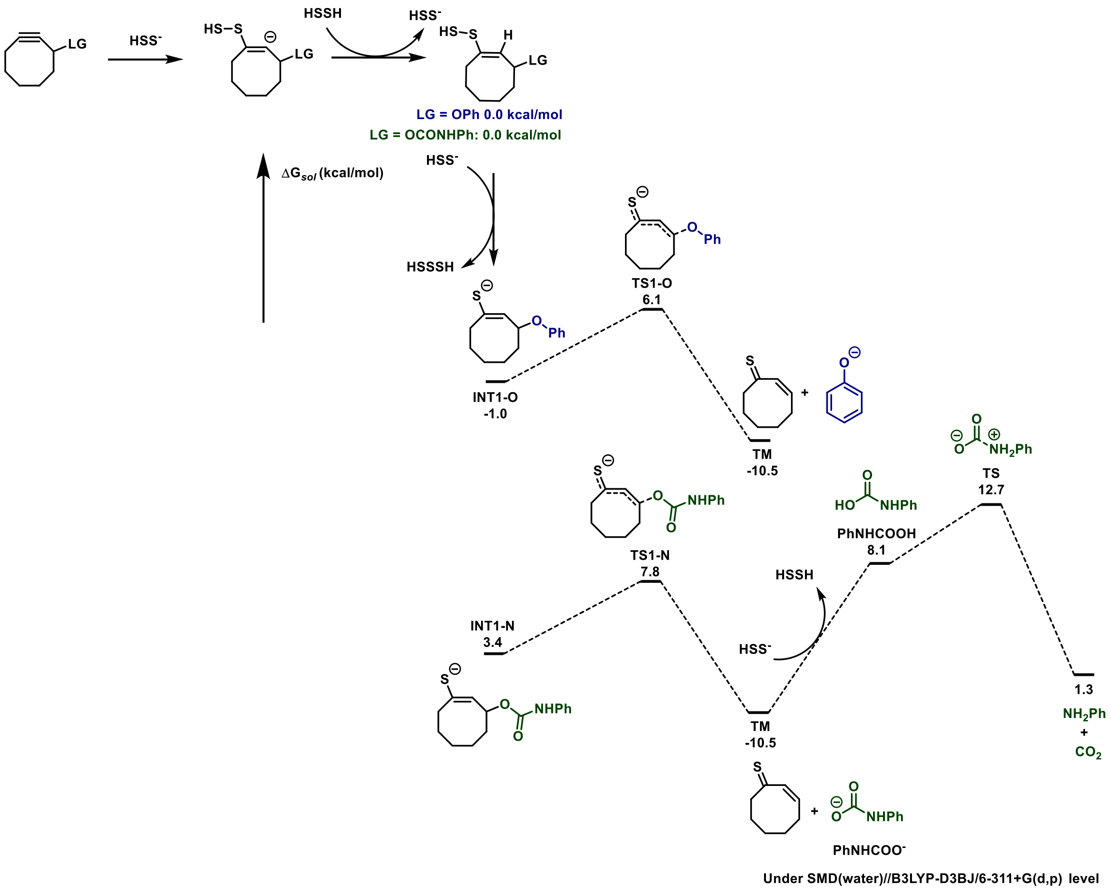

Figure S4. Computational reaction models and the calculated Gibbs free energies and activation free energies, used to investigate the difference in reaction rates between probes with an ether linker versus a carbamate linker. The *O*-derived payload is colored blue, and the *N*-derived payload is colored green. Computated under SMD(water)//B3LYP-D3BJ/6-311+G(d,p) level.

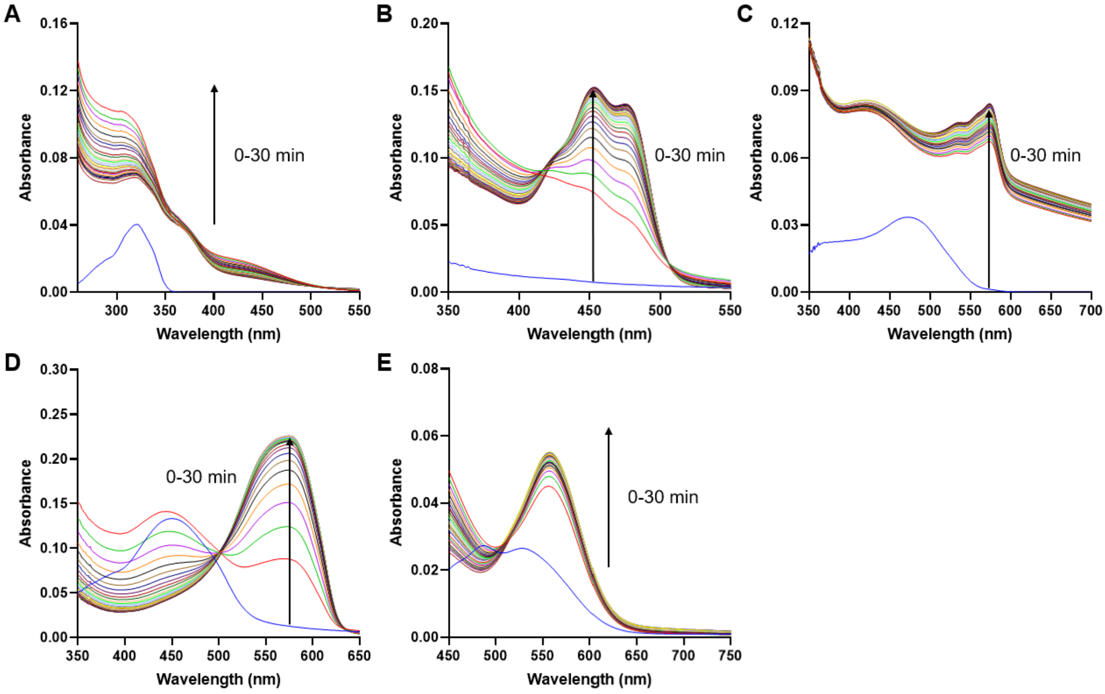

Figure S5. The UV–vis spectroscopy data of the ether-based probes **Cyne-1~5** when treated with Na_2S_2.

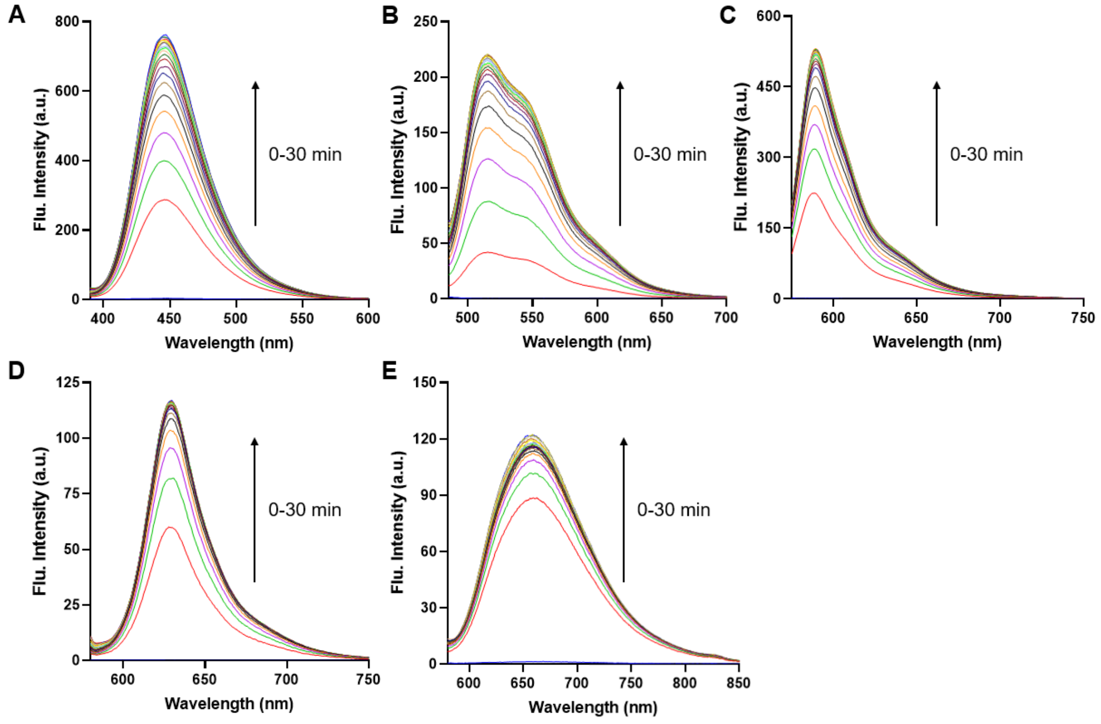

Figure S6. The fluorescence spectroscopy data of the ether-based probes **Cyne-1~5** when treated with Na_2S_2. E_x/E_m please see Table 1. Slit width: 5/5 nm.

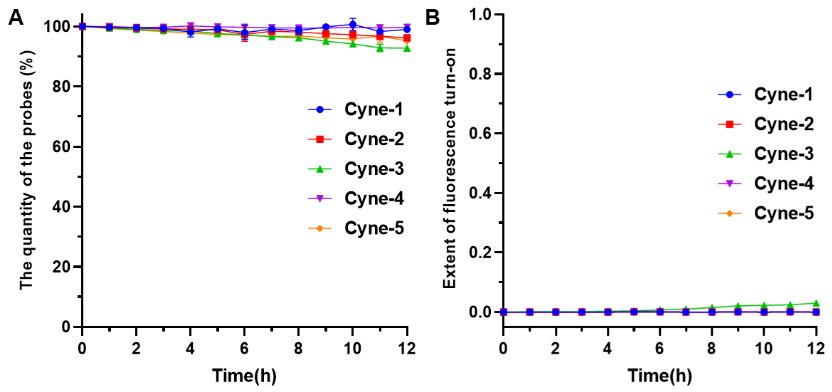

Figure S7. Stability evaluation of probes **Cyne-1~5**. A) High-performance liquid chromatography (HPLC) was employed to quantify the residual amount of the probes. The initial value (100%) was defined by the peak area at t = 0. B) Fluorescence Spectrophotometer was utilized to examine the fluorescence turn-on of the probes. The initial value (0.0) was defined by the fluorescence intensity at t = 0, while the upper limit (1.0) was set as the fluorescence intensity when the probe fluorescence was fully activated. Test conditions: probes: 1 μmol/L; Na_2S_2: 1 μM. The error bars represent standard deviations (s.d.) from three independent experiments. E_x/E_m please see Table 1. Slit width: 5/5 nm.

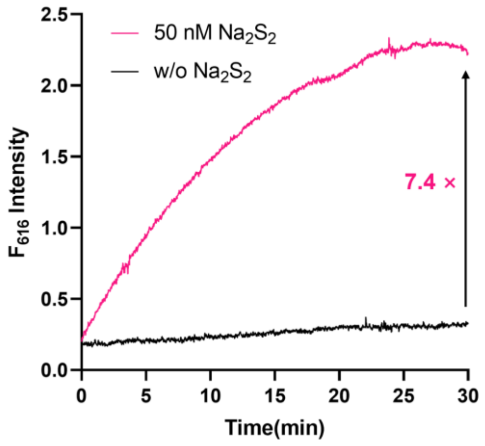

Figure S8. The probe **Cyne-4** (1 μmol/L) is utilized for the detection of trace amounts of H_2S_2/H_2S_n (50 nmol/L).

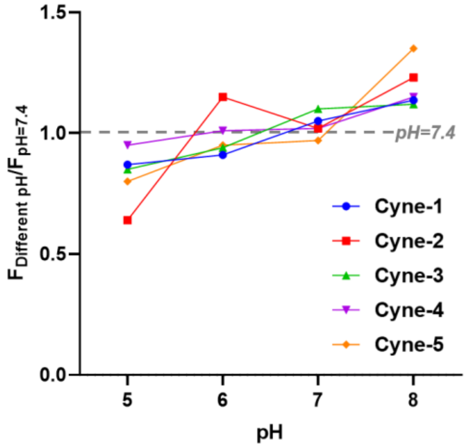

Figure S9. The fluorescent performance of probes **Cyne-1~5** under different pH conditions. E_x/E_m please see Table 1. Slit width: 5/5 nm.

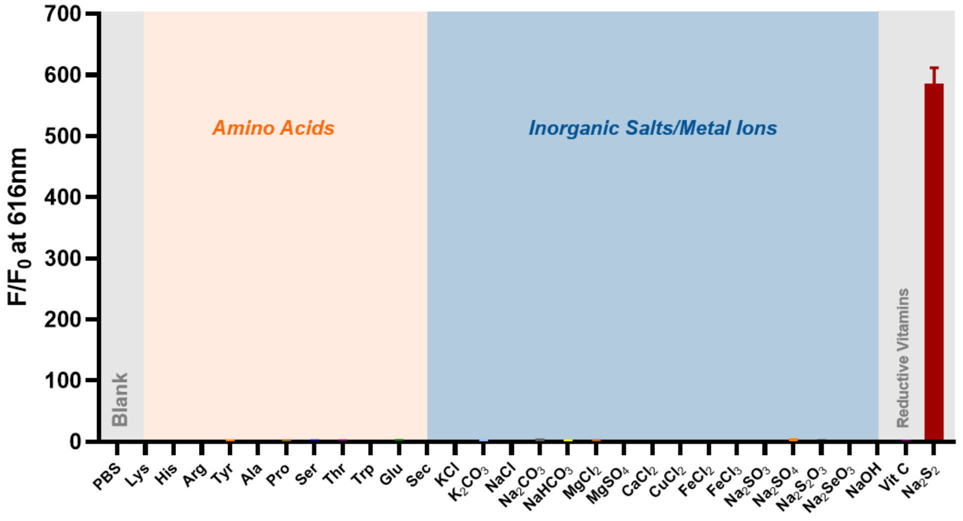

Figure S10. F/F_0 of probe **Cyne-4** (10 μmol/L) at E_m = 616 nm in the presence of various amino acids, inorganic salts, metal ions and reduced small molecule compounds commonly found *in vivo*. All analytes were at 100

μmol/L concentration. The error bars represent standard deviations (s.d.) from three independent experiments. E_x/E_m at 549/616 nm. Slit width: 5/5 nm.

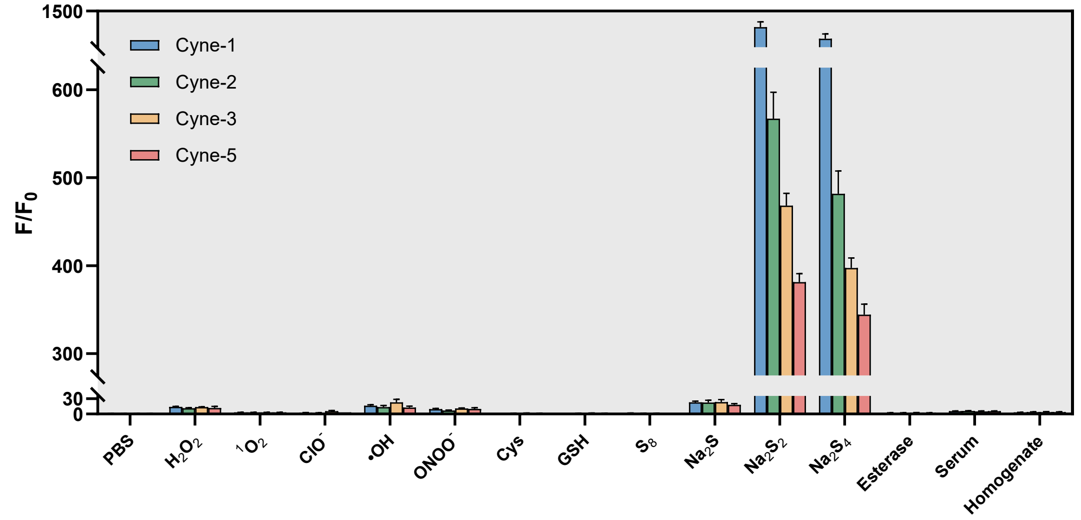

Figure S11. Fold change in fluorescence of the **Cyne** probe series (10 μmol/L) upon treatment with common ROS, RNS, RSS, and selected biological fluids. All analytes were at 100 μmol/L concentration. The error bars represent standard deviations (s.d.) from three independent experiments.

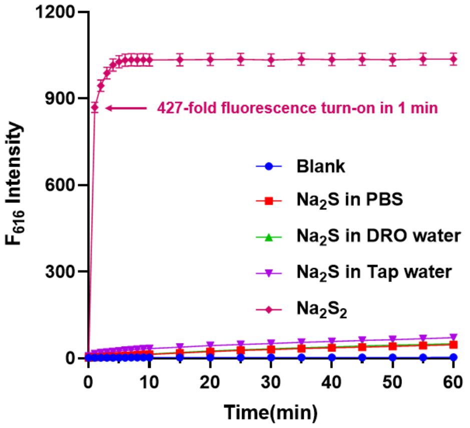

Figure S12. Comparison of fluorescence turn-on of probe in Na_2S or Na_2S_2. Test conditions: probe **Cyne-4**: 10 μmol/L; Na_2S or Na_2S_2: 100 μmol/L. **Note**: The addition of 10 eq. of Na_2S_2 is for a rapid reaction to determine the maximum fluorescence intensity that 10 μmol/L probe can cause. This fluorescence intensity represents the maximum level of fluorescence turn-on caused by 10 μmol/L Na_2S_2. The error bars represent standard deviations (s.d.) from three independent experiments. E_x/E_m at 549/616 nm. Slit width: 5/5 nm.

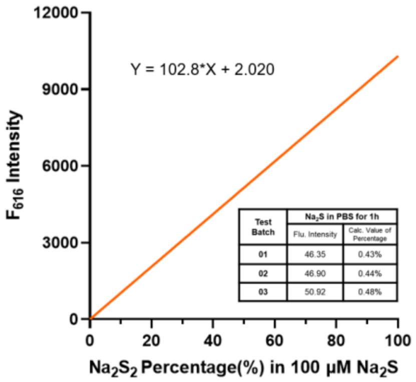

Figure S13. Calculate the proportion of H_2S_2 in 100 μmol/L Na_2S solution. The linear regression equation is determined based on the maximum fluorescence onset caused by 100 μM Na_2S_2 and the background of the probe. E_x/E_m at 549/616 nm. Slit width: 5/5 nm.

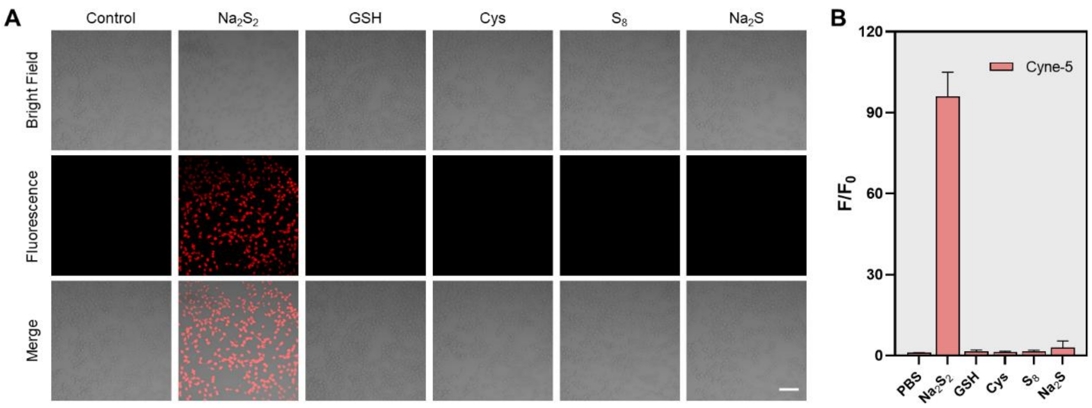

Figure S14. The selectivity of the probe **Cyne-5** for visualizing H_2S_n in 4T1 cells. 4T1 cells were incubated with the probe **Cyne-5** (2 μmol/L) for 30 min, then washed by PBS buffer and treated with various analytes (10 μmol/L). Images were acquired after 15 min. Scale bar represents 100 μm in all images.

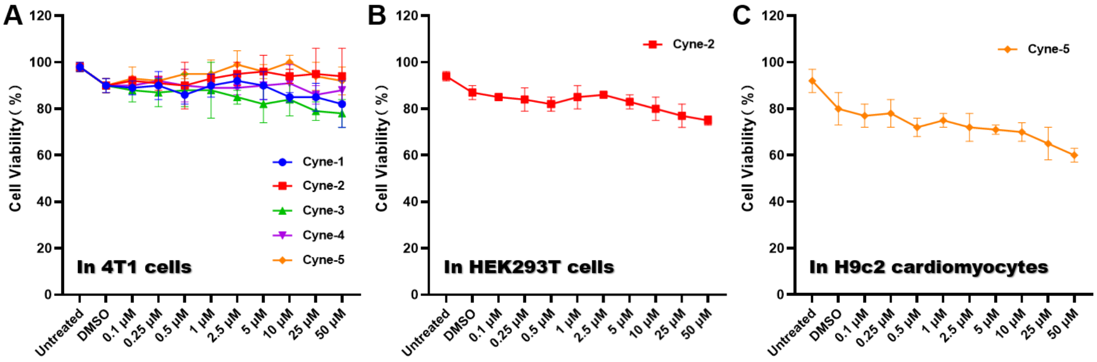

Figure S15. Cytotoxicity of probes **Cyne-1~5** in 4T1 cells, HEK293T cells, or H9c2 cardiomyocytes. Cells were incubated with increasing amounts of probes **Cyne-1~5** for 24 hours. The probes exhibited negligible cytotoxicity

after 24 hours incubation. The error bars represent standard deviations (s.d.) from three independent CCK-8 assays.

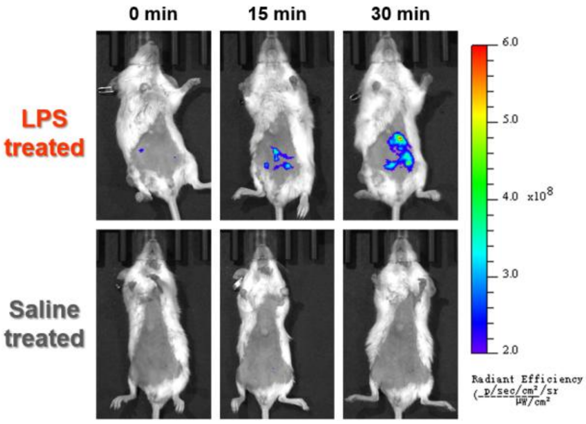

Figure S16. *In vivo* imaging of endogenous H_2S_n in BALB/c mice. Mice were intraperitoneally administered 100 μL of lipopolysaccharide (2 mg/mL) or normal saline. Twenty-four hours later, each mouse was intraperitoneally administered 100 μL of the probe **Cyne-5** (10 μmol/L in PBS buffer, containing 0.1% DMSO). Imaging was performed using the PerkinElmer IVIS® Lumina LT Series III *in vivo* imaging system. Excitation filter: 570 nm; Emission filter: 660 nm.

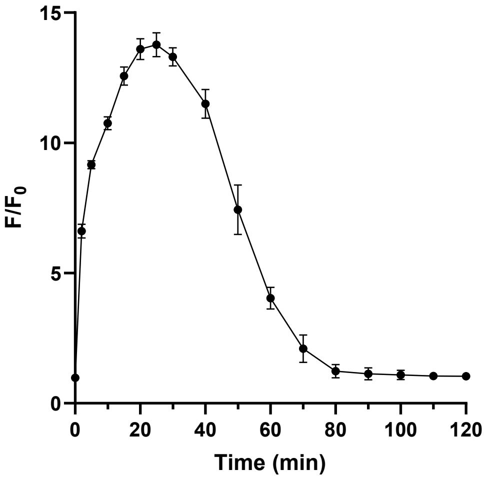

Figure S17. Quantification of fluorescence intensity over 120 min in BALB/C mice following intraperitoneal injection of the probe **Cyne-5** and subsequent Na_2S_2.

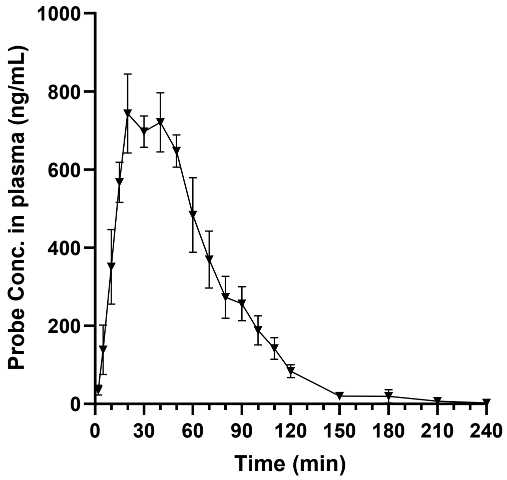

Figure S18. Pharmacokinetic analysis of the probe **Cyne-5** concentration in mice plasma.

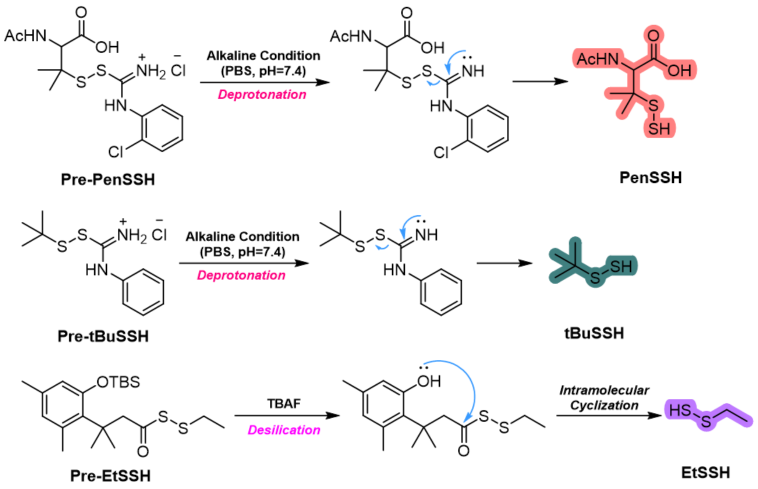

Figure S19. The mechanisms of selected three persulfide donors releasing corresponding persulfides.

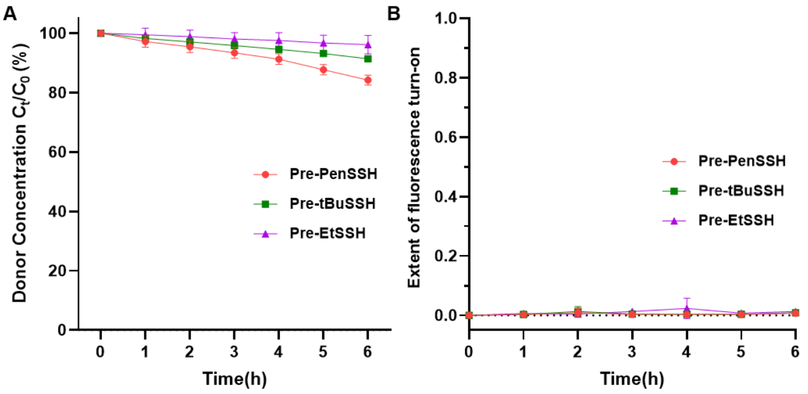

Figure S20. Stability evaluation of the donors **Pre-PenSSH**, **Pre-tBuSSH** and **Pre-EtSSH**. A) High-performance liquid chromatography (HPLC) was employed to quantify the residual amount of the donors. The initial value

(100%) was defined by the peak area at t = 0. **Note**: Due to the rapid release of corresponding persulfides by **Pre-PenSSH** and **Pre-tBuSSH** under weakly alkaline conditions, their stability was tested in a potassium hydrogen phthalate buffer at pH=4. B) Fluorescence Spectrophotometer was utilized to examine the fluorescence turn-on of the probe that was induced by the donors. The initial value (0.0) was defined by the fluorescence intensity at t = 0, while the upper limit (1.0) was set as the fluorescence intensity when the probe fluorescence was fully activated. Test conditions: probe **Cyne-4**: 10 μmol/L; persulfide donors: 50 μmol/L. The error bars represent standard deviations (s.d.) from three independent experiments. E_x/E_m at 549/616 nm. Slit width: 5/5 nm.

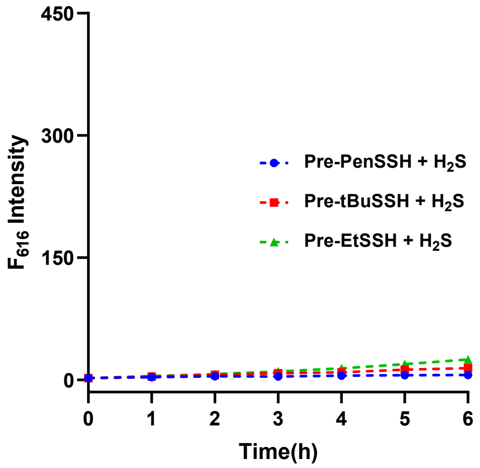

Figure S21. Evaluation of the stability of persulfide donors when co-incubated with H_2S without the addition of the trigger. The error bars represent standard deviations (s.d.) from three independent experiments. E_x/E_m at 549/616 nm. Slit width: 5/5 nm.

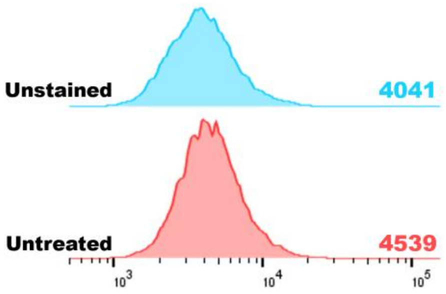

Figure S22. Corresponding to the unstained group and the untreated group in **Figure 8G-I**. The numbers in the figure represent the mean fluorescence intensity. E_x wavelength: 488 nm; E_m channel: 530/30 nm. Results are representative of three independent experiments.

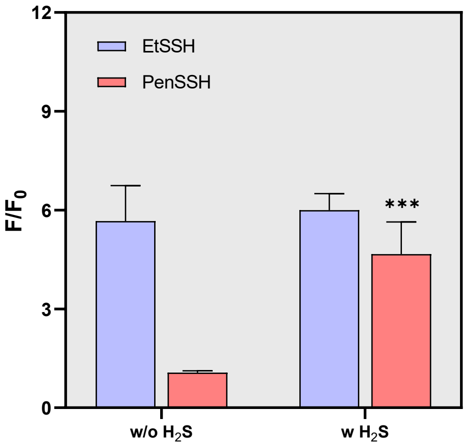

Figure S23. Disproportionation of persulfides (20 μmol/L) with varying steric hindrance: Flow cytometric comparison of H_2S_2/H_2S_n levels using probe **Cyne-4** (5 μmol/L), with or without Na_2S (20 μmol/L).

Table S1. Comparison of probe **Cyne-5** with other reported fluorescent probes for H_2S_2/H_2S_n.

Probe structure	E_m (nm)	Time to plateau (min)	Spectroscopic testing conditions	LOD (nM)	Reference
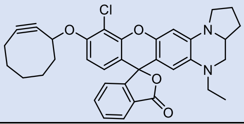	660	3	1 μM probe<br>1 μM Na_2S_2	3.3	This work
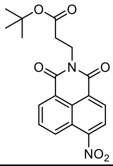	540	Not measured	10 μM probe<br>200 μM Na_2S_2	4800	Yoon et.al., *Sensor Actuat. B-Chem.* **2020**, *322*, 128564.
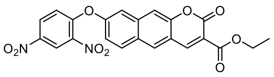	430/530	> 60	10 μM probe<br>100 μM Na_2S_2	3010	Chen et.al., *Talanta* **2024**, *268*, 125293.
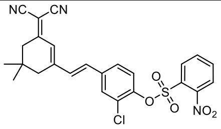	675	30	10 μM probe<br>80 μM Na_2S_4	284	Yu et.al., *Anal. Chem.* **2025**, *97*, 14265-14272.
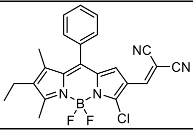	740	30	10 μM probe<br>100 μM Na_2S_4	293	Zhao et.al., *ACS Nano* **2023**, *17*, 22060-22070
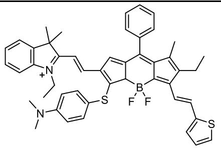	920	4	10 μM probe<br>100 μM Na_2S_4	105	Gu et.al., *Anal. Chem.* **2024**, *96*, 20049-20056.
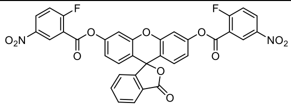	515	5	10 μM probe<br>50 μM Na_2S_2	71	Xian et.al., *J. Am. Chem. Soc.* **2014**, 136, 7257- 7260.

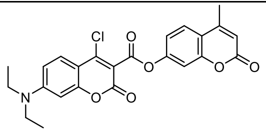	461/510	30	10 µM probe<br>100 µM Na_2S_2	65	Song et.al., *Sensor Actuat. B-Chem.* **2018**, *258*, 125-132

Compound	$t_{1/2}$(min)	Peak time of H_2S (min)	Peak concentration of H_2S (µM)
**ZYSA03**	7.1 ± 0.1	20.0 ± 9.0	124.0 ± 21.0
**ZYSH09**	243.5 ± 13.2	162.4 ± 1.2	57.4 ± 0.9

Table S2. Characteristics of the decomposition of two thiocarboxylic acids in the presence of PLE (3 units/mL) in PBS at 37 °C. (Reference from our previous work: *Journal of Medicinal Chemistry* **2024** *67* (19), 17350-17362)

10. NMR spectrum and HRMS data

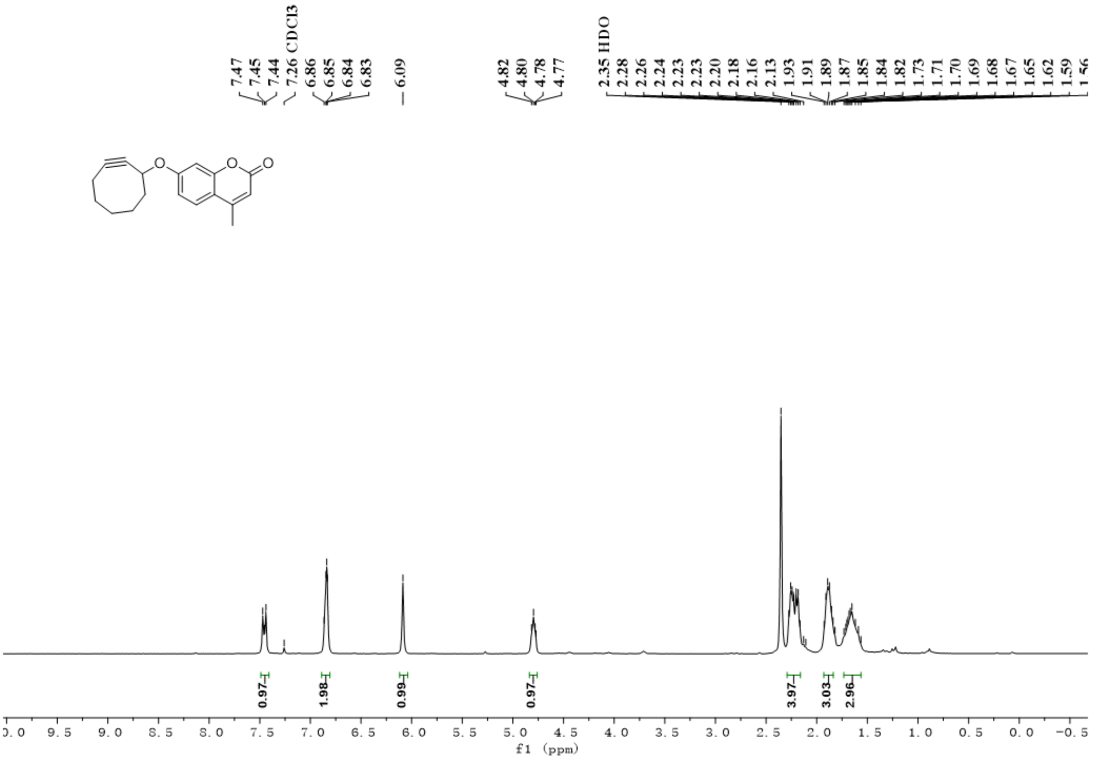

^{1}H NMR of compound **Cyne-1** (300 MHz, $CDCl_3$)

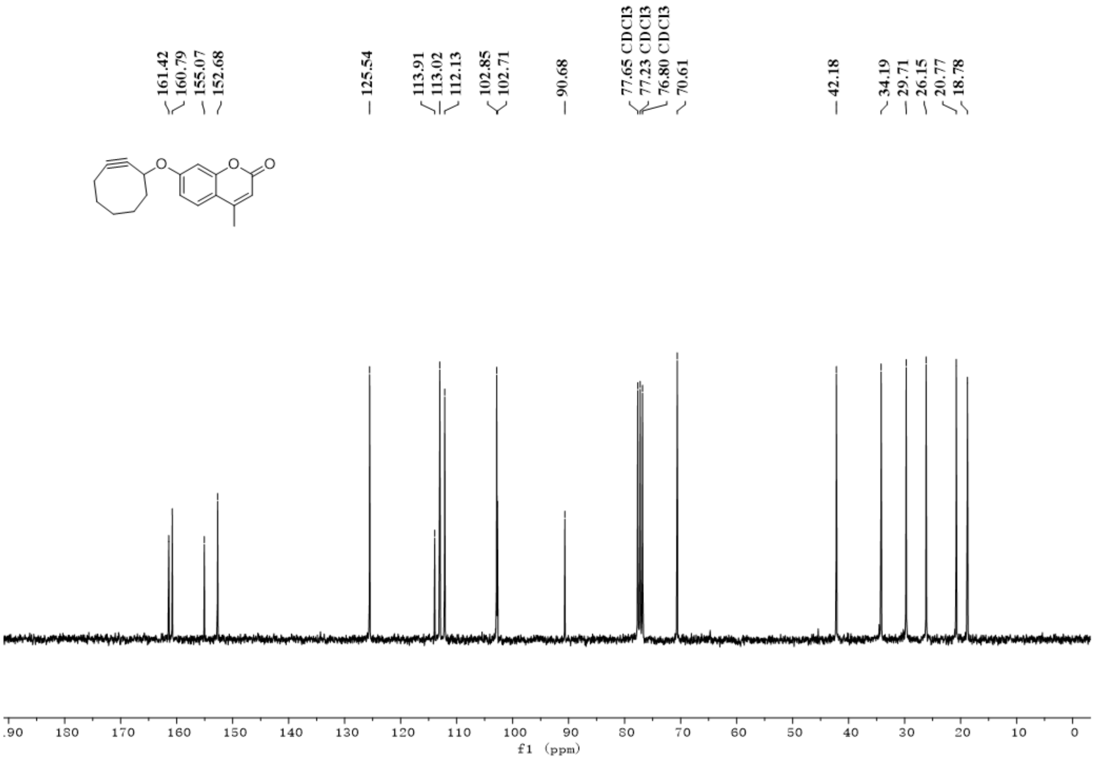

^{13}C NMR of compound **Cyne-1** (75 MHz, $CDCl_3$)

Spectra

Fragmentor Voltage	Collision Energy	Ionization Mode
120	0	ESI

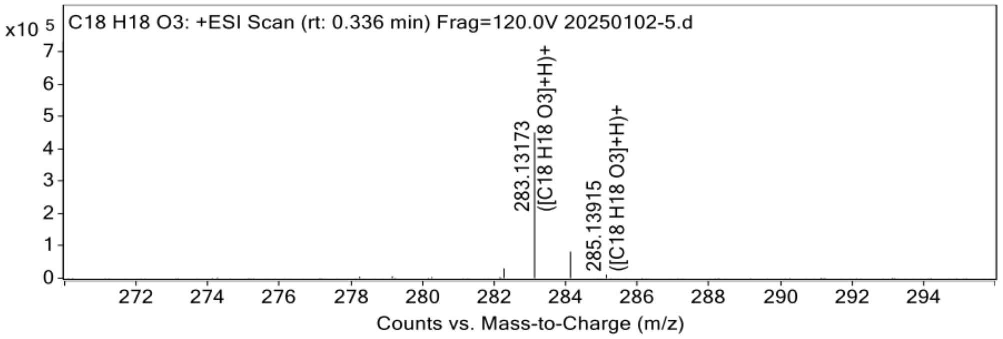

Peak List

m/z	z	Abund	Formula	Ion
64.01588		254375.64		
283.13173	1	450772.75	C18 H18 O3	(M+H)+
565.25567	1	196185.86		
587.23871	1	808236.25		
588.24094	1	327134.19		

Formula Calculator Element Limits

Element	Min	Max
C	10	30
H	10	30
O	2	4

Formula Calculator Results

Formula	Best	Mass	Tgt Mass	Diff (ppm)	Ion Species	CalculatedMz
C18 H18 O3	TRUE	282.12447	282.12559	4	C18 H19 O3	283.13287

HRMS(ESI) of compound **Cyne-1**

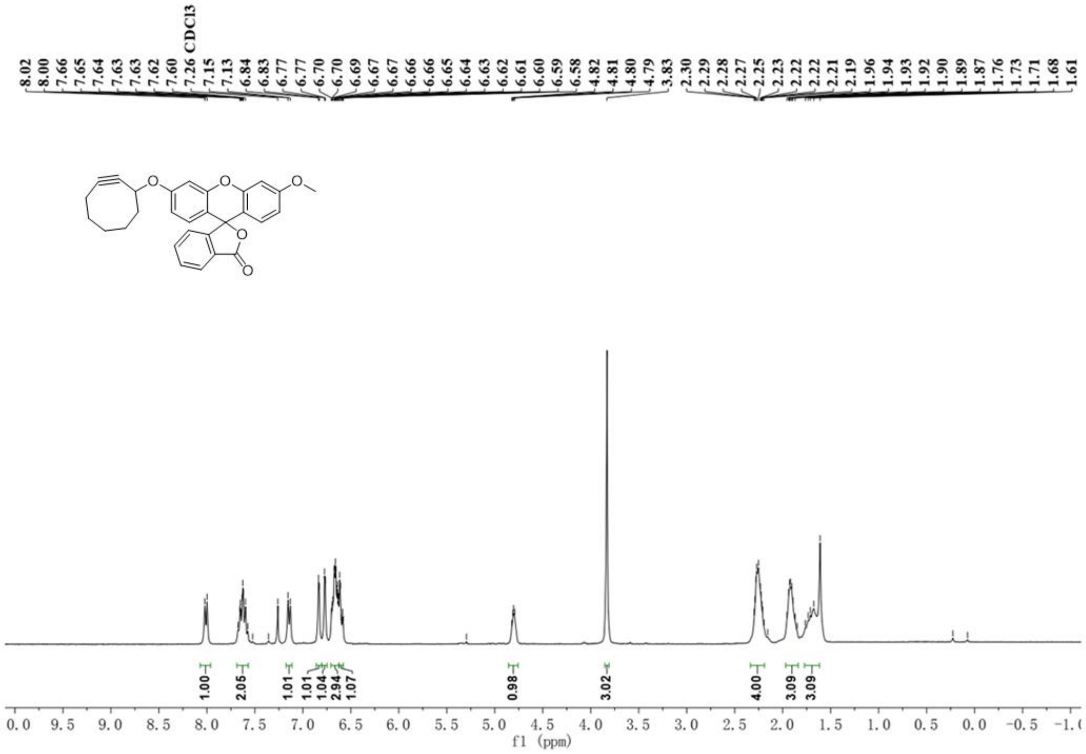

^{1}H NMR of compound **Cyne-2** (300 MHz, $CDCl_3$)

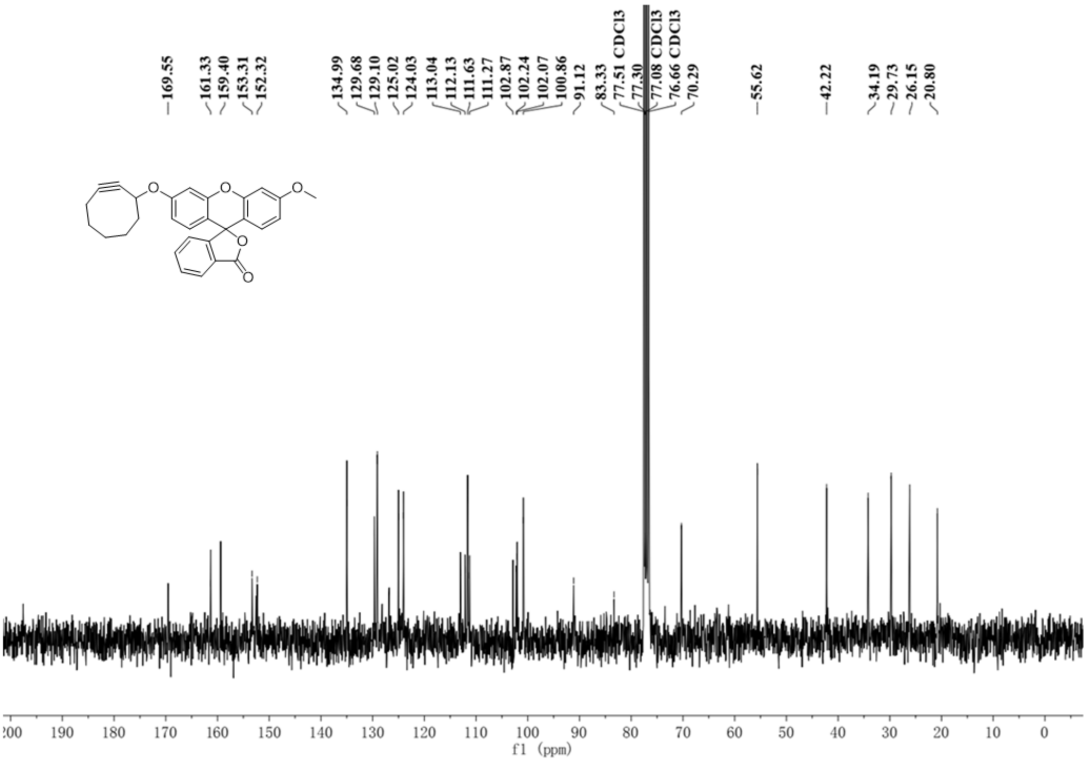

^{13}C NMR of compound **Cyne-2** (75 MHz, $CDCl_3$)

Spectra

Fragmentor Voltage	Collision Energy	Ionization Mode
120	0	ESI

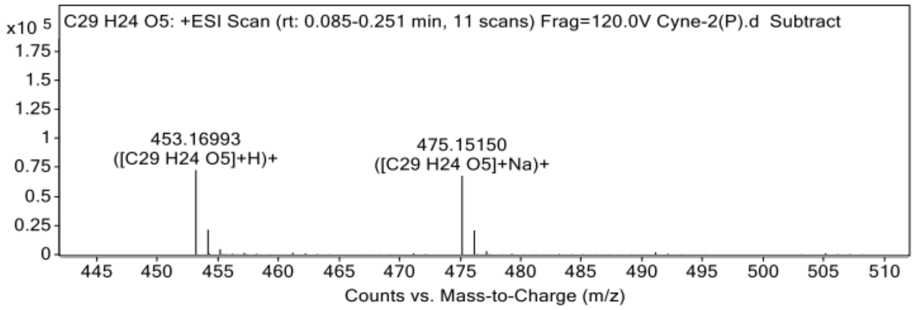

Peak List

m/z	z	Abund	Formula	Ion
361.10717	1	130869.88		
453.16993	1	72599.72	C29 H24 O5	(M+H)+
835.25131	1	96167.23		
927.31458	1	305255.56		
928.31766	1	190385.05		

Formula Calculator Element Limits

Element	Min	Max
C	29	29
H	24	24
O	5	5
N	0	1

Formula Calculator Results

Formula	Best	Mass	Tgt Mass	Diff (ppm)	Ion Species	CalculatedMz
C29 H24 O5	TRUE	452.16255	452.16237	-0.4	C29 H25 O5	453.16965
C29 H24 O5	TRUE	452.16358	452.16237	-2.67	C29 H24 Na O5	475.15159

HRMS(ESI) of compound **Cyne-2**

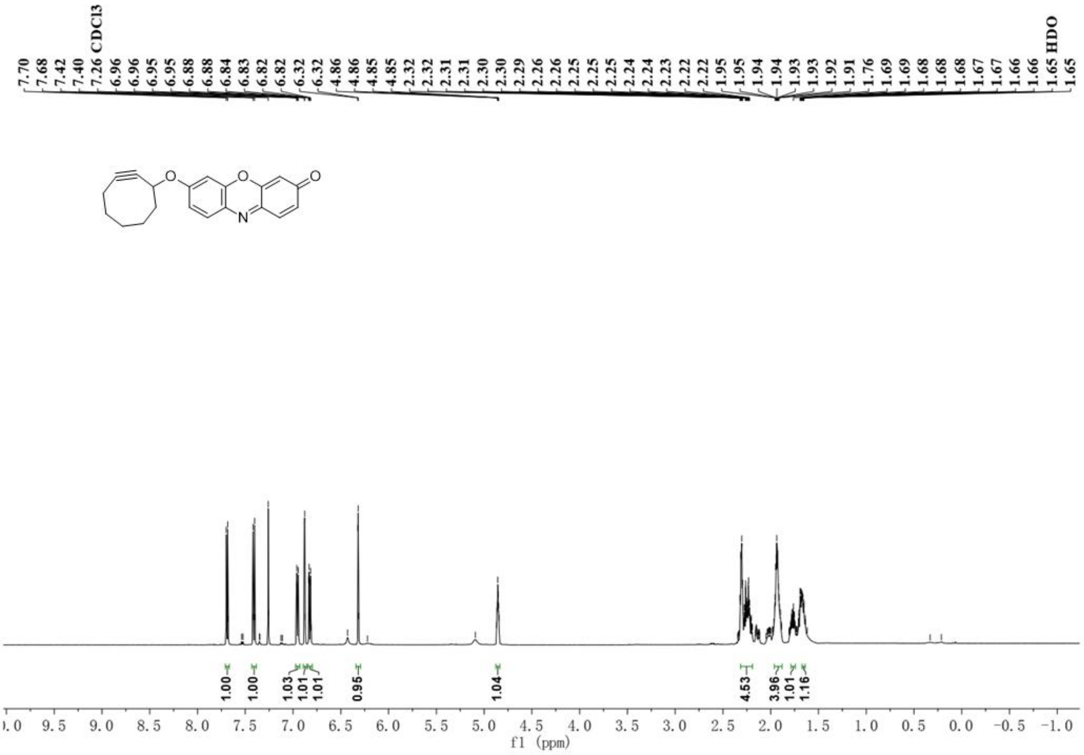

^{1}H NMR of compound **Cyne-3** (600 MHz, $CDCl_3$)

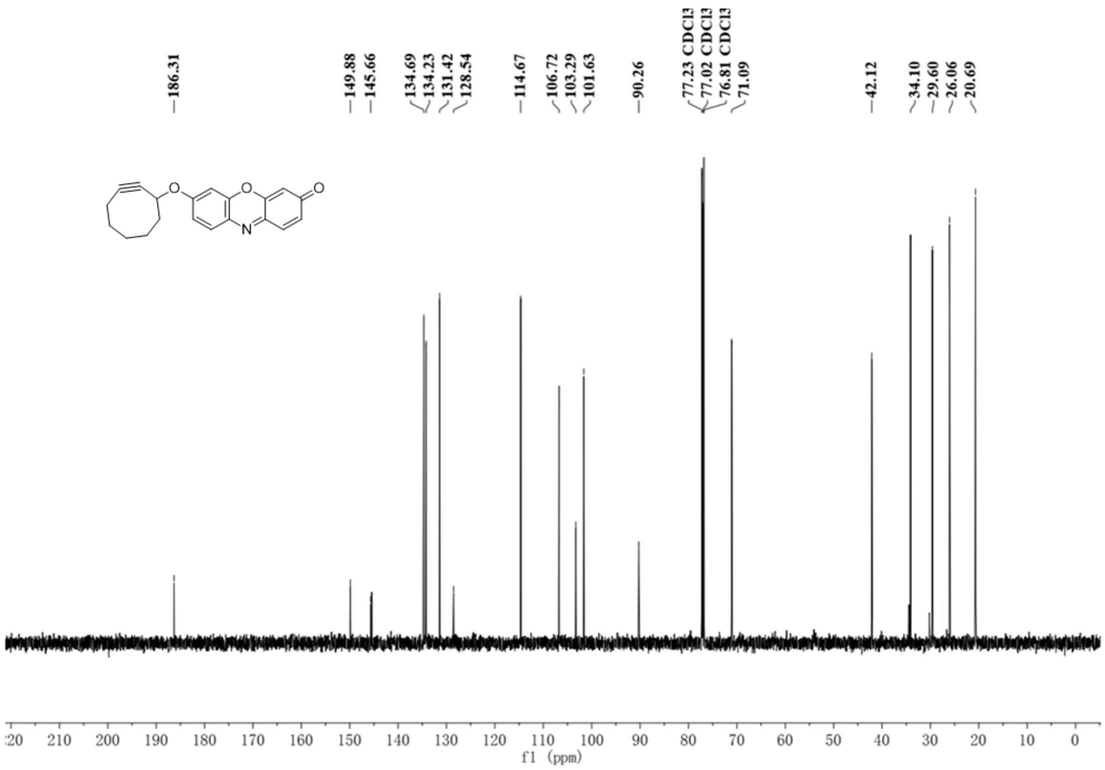

^{13}C NMR of compound **Cyne-3** (151 MHz, $CDCl_3$)

Spectra

Fragmentor Voltage	Collision Energy	Ionization Mode
120	0	ESI

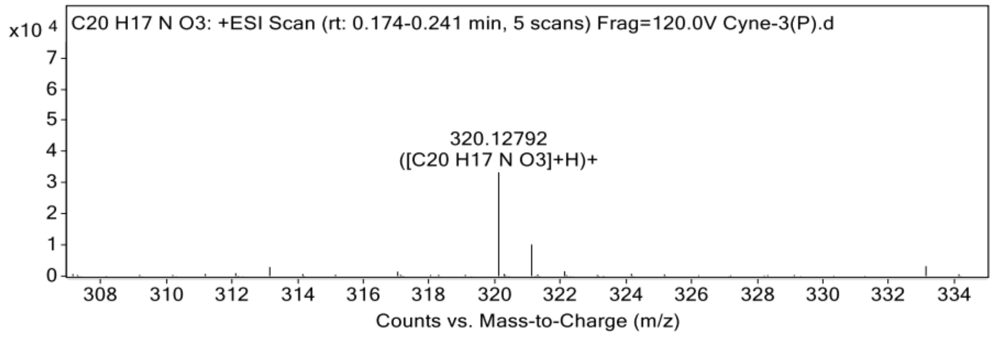

Peak List

m/z	z	Abund
279.09403	1	594058
283.16551	1	215277.09
557.18254	1	242445.09
579.16279	1	2316049
580.16636	1	909274.63

Formula Calculator Element Limits

Element	Min	Max
C	20	20
H	17	17
O	3	3
N	0	1

Formula Calculator Results

Formula	Best	Mass	Tgt Mass	Diff (ppm)	Ion Species	CalculatedMz
C20 H17 N O3	TRUE	319.12051	319.12084	1.05	C20 H18 N O3	320.12812

HRMS(ESI) of compound **Cyne-3**

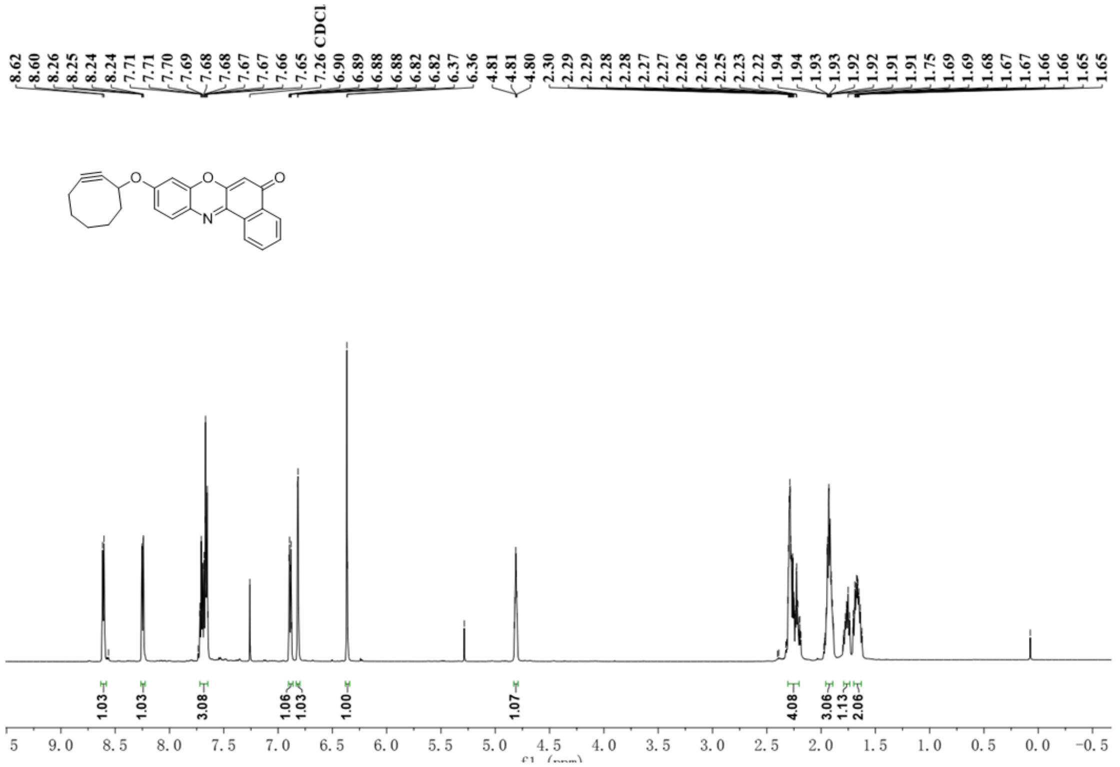

^{1}H NMR of compound **Cyne-4** (600 MHz, $CDCl_3$)

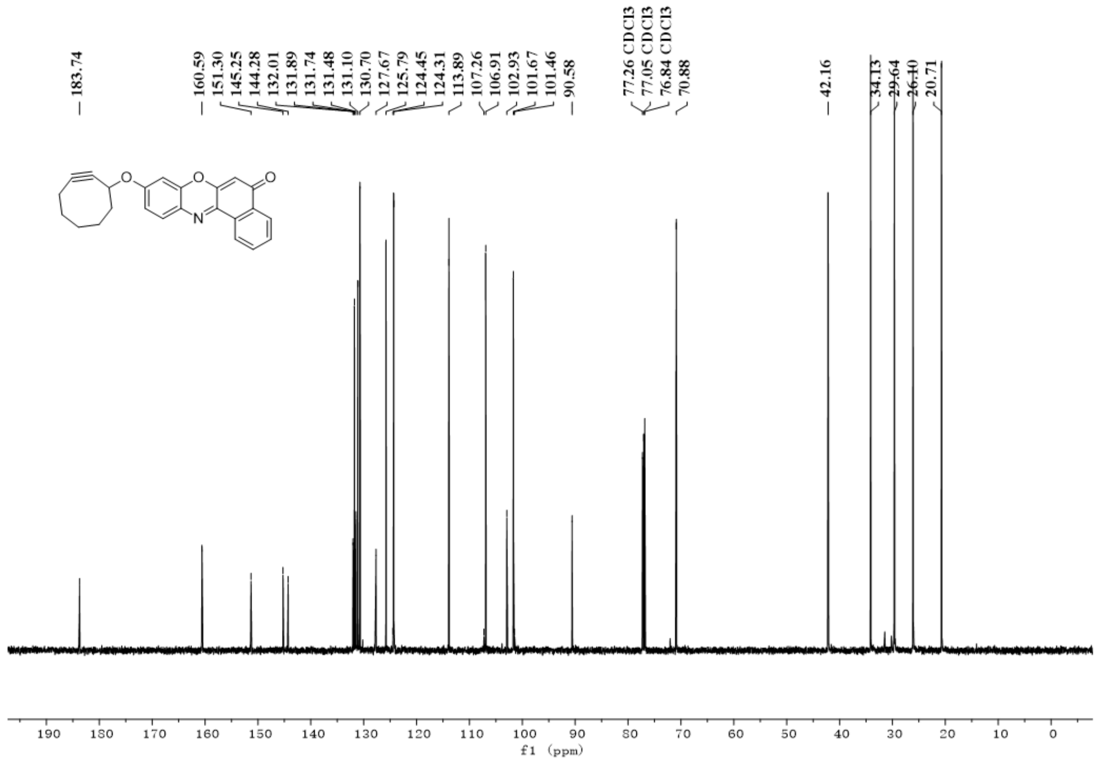

^{13}C NMR of compound **Cyne-4** (151 MHz, $CDCl_3$)

Spectra

Fragmentor Voltage	Collision Energy	Ionization Mode
120	0	ESI

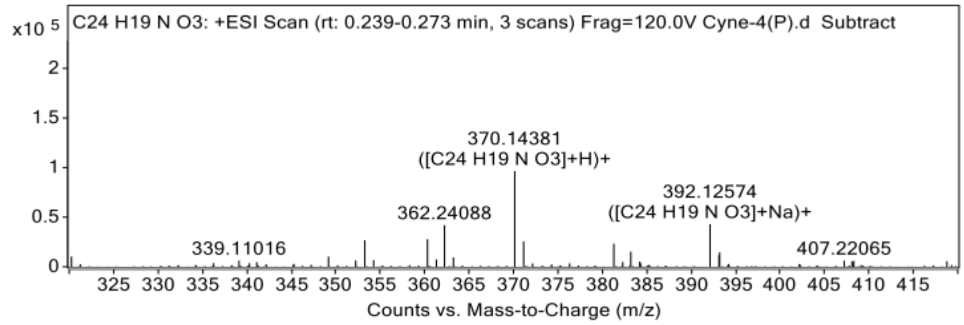

Peak List

m/z	z	Abund
304.26126	1	188474.8
701.49393	1	421024.5
761.26282	1	521172.63
762.26579	1	263150.69
814.57797	1	351868.38

Formula Calculator Element Limits

Element	Min	Max
C	24	24
H	19	19
O	3	3
N	0	1

Formula Calculator Results

Formula	Best	Mass	Tgt Mass	Diff (ppm)	Ion Species	CalculatedMz
C24 H19 N O3	TRUE	369.13664	369.13649	-0.41	C24 H20 N O3	370.14377
C24 H19 N O3	TRUE	369.13658	369.13649	-0.22	C24 H19 N Na O3	392.12571

HRMS(ESI) of compound **Cyne-4**

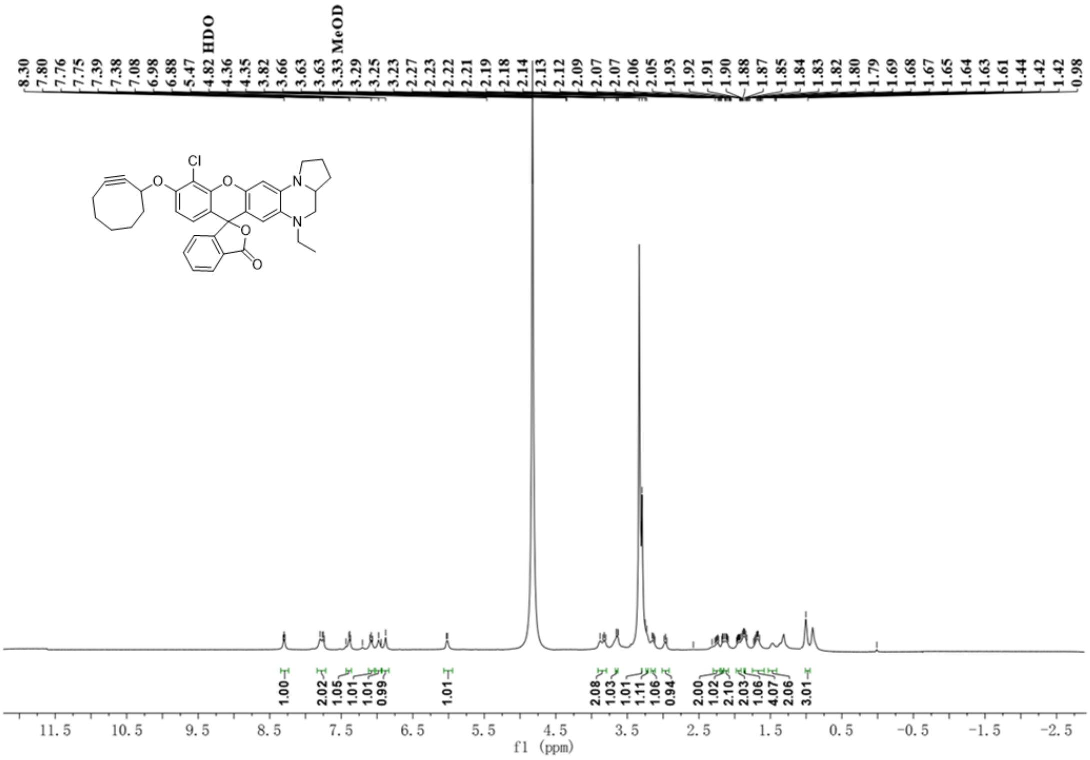

^{1}H NMR of compound **Cyne-5** (600 MHz, MeOD)

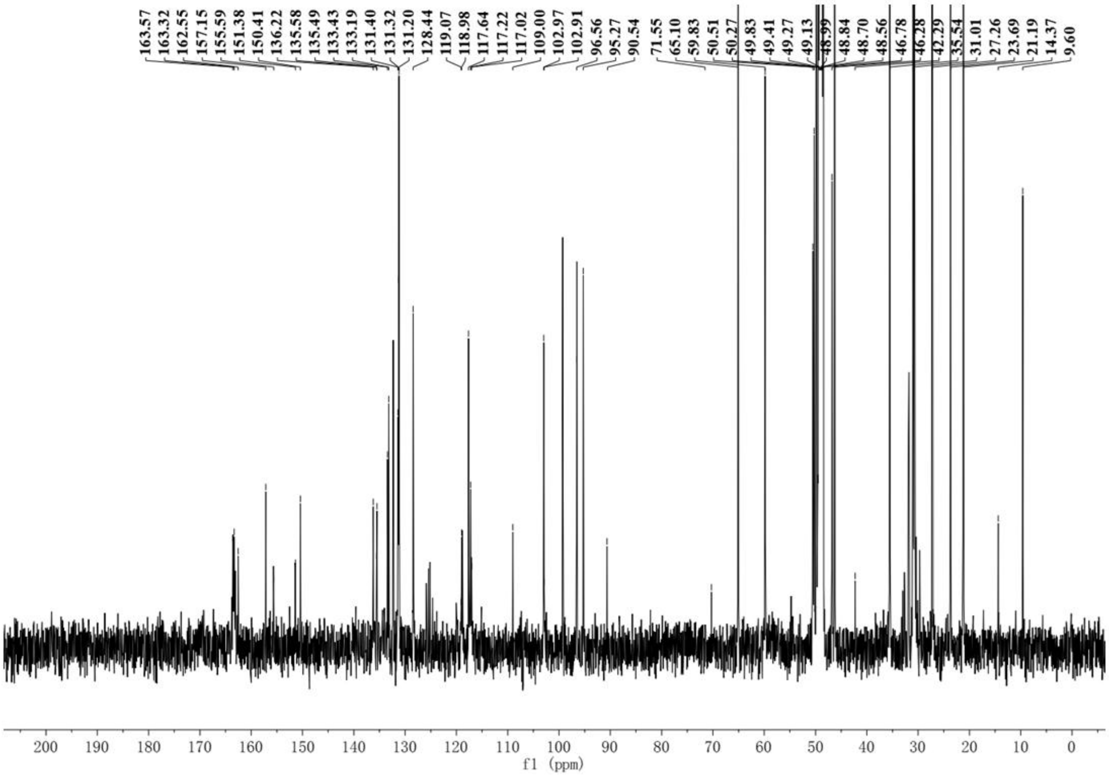

^{13}C NMR of compound **Cyne-5** (151 MHz, MeOD)

Spectra

Fragmentor Voltage	Collision Energy	Ionization Mode
120	0	ESI

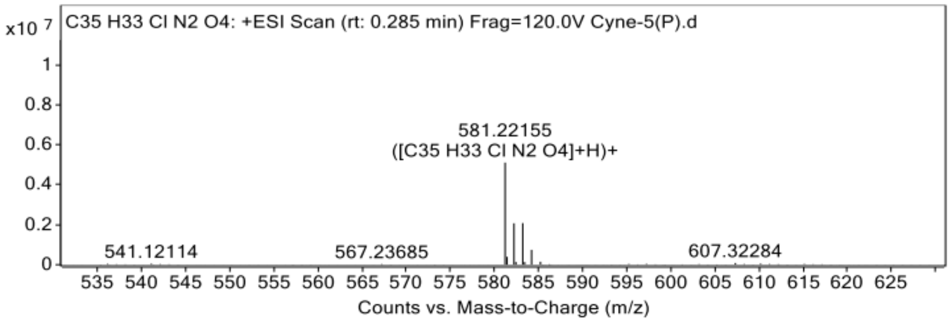

Peak List

m/z	z	Abund	Formula	Ion
304.26349	1	482505.16		
581.22155	1	5106553	C35 H33 Cl N2 O4	(M+H)+
582.22475	1	2070145.75	C35 H33 Cl N2 O4	(M+H)+
583.22069	1	2080657.13	C35 H33 Cl N2 O4	(M+H)+
584.22201	1	742169.44	C35 H33 Cl N2 O4	(M+H)+

Formula Calculator Element Limits

Element	Min	Max
C	35	35
H	33	33
O	4	4
N	2	2
Cl	0	1

Formula Calculator Results

Formula	Best	Mass	Tgt Mass	Diff (ppm)	Ion Species	CalculatedMz
C35 H33 Cl N2 O4	TRUE	580.21426	580.21289	-2.37	C35 H34 Cl N2 O4	581.22016

HRMS(ESI) of compound **Cyne-5**

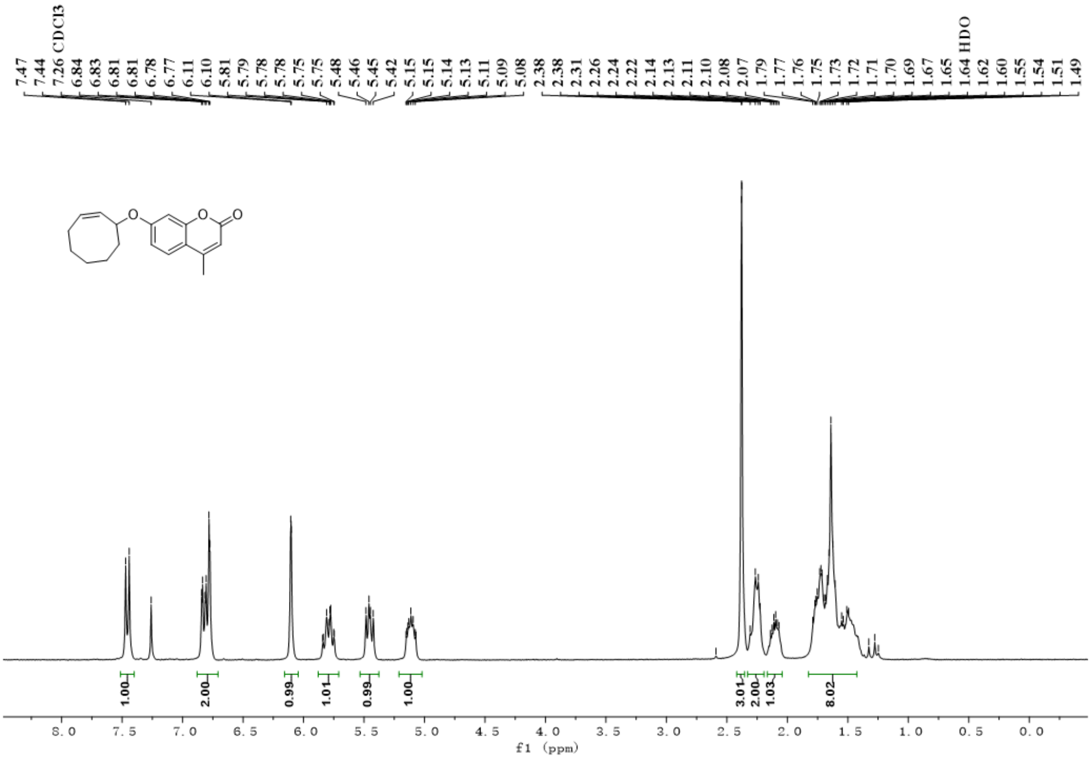

^{1}H NMR of compound **Cene-1** (300 MHz, $CDCl_3$)

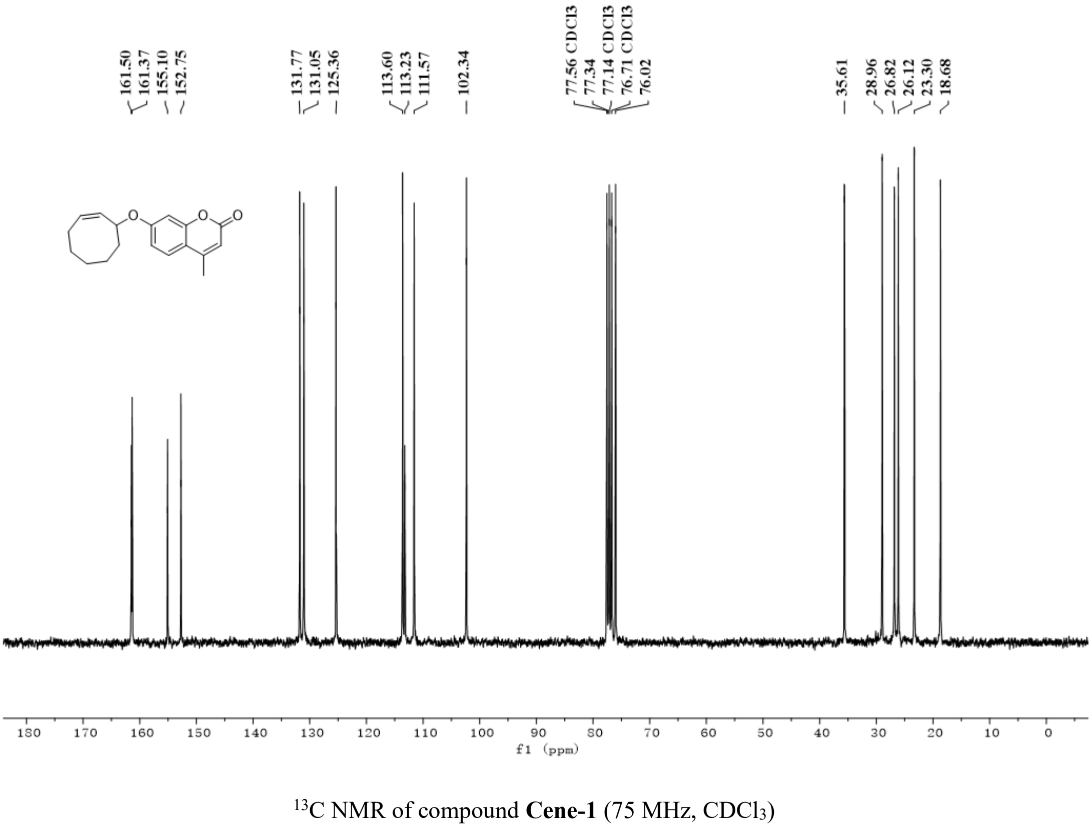

^{13}C NMR of compound **Cene-1** (75 MHz, $CDCl_3$)

Spectra

Fragmentor Voltage	Collision Energy	Ionization Mode
175	0	ESI

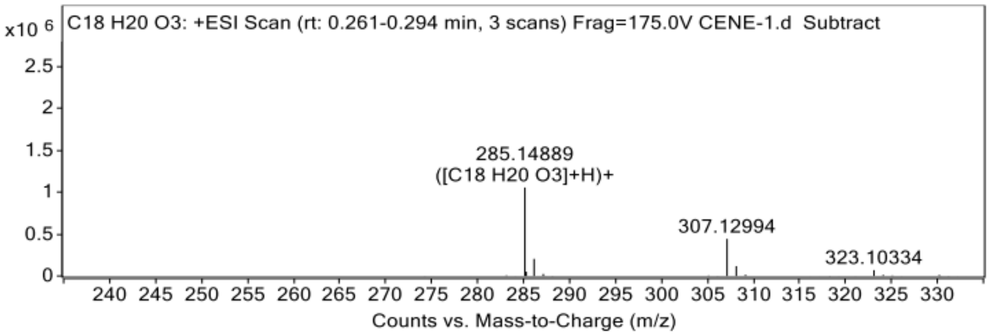

Peak List

m/z	z	Abund	Formula	Ion
285.14889	1	1055124.25	C18 H20 O3	(M+H)+
307.12994	1	442853.94		
591.27123	1	4473262		
592.27467	1	1811917.5		
699.36384		487481.13		

Formula Calculator Element Limits

Element	Min	Max
C	8	28
H	10	30
O	2	4

Formula Calculator Results

Formula	Best	Mass	Tgt Mass	Diff (ppm)	Ion Species	CalculatedMz
C18 H20 O3	TRUE	284.14144	284.14124	-0.69	C18 H21 O3	285.14852

HRMS(ESI) of compound **Cene-1**

11. References in *Supporting Information*

1. Kang, D.; Kim, J., Bioorthogonal Retro-Cope Elimination Reaction of *N,N*-Dialkylhydroxylamines and Strained Alkynes. *J. Am. Chem. Soc.* **2021,** *143* (15), 5616-5621.

2. Mugherli, L.; Burchak, O. N.; Chatelain, F.; Balakirev, M. Y., Fluorogenic ester substrates to assess proteolytic activity. *Bioorg. Med. Chem. Lett.* **2006,** *16* (17), 4488-4491.

3. Xu, L.; Chu, H.; Gao, D.; Wu, Q.; Sun, Y.; Wang, Z.; Ma, P.; Song, D., Chemosensor with ultra-high fluorescence enhancement for assisting in diagnosis and resection of ovarian cancer. *Anal. Chem.* **2023,** *95* (5), 2949-2957.

4. Chen, W.; Xu, S.; Day, J. J.; Wang, D.; Xian, M., A general strategy for development of near-infrared fluorescent probes for bioimaging. *Angew. Chem. Int. Ed.* **2017,** *129* (52), 16838-16842.

5. Tong, X.; Chen, J.; Wang, M.; Liu, J.; Li, J.; Wang, X.; Zuo, Y.; Xu, X.; Wang, Y.; Wang, B.; Guo, W.; Zheng, Y., Development of a Bioorthogonal Click-to-Release Reaction for Hydrogen Polysulfide (H_2S_n) Detection. *Anal. Chem.* **2024**. *96*(39), 15631–15639

6. Kosiova, I.; Kovackova, S.; Kois, P., Synthesis of coumarin–nucleoside conjugates via

Huisgen 1, 3-dipolar cycloaddition. *Tetrahedron.* **2007,** *63* (2), 312-320.

7. Khodade, V. S.; Toscano, J. P., Development of S-substituted thioisothioureas as efficient hydropersulfide precursors. *J. Am. Chem. Soc.* **2018,** *140* (50), 17333-17337.

8. Khodade, V. S.; Liu, Q.; Zhang, C.; Keceli, G.; Paolocci, N.; Toscano, J. P., Arylsulfonothioates: Thiol-Activated Donors of Hydropersulfides which are Excreted to Maintain Cellular Redox Homeostasis or Retained to Counter Oxidative Stress. *J. Am. Chem. Soc.* **2025,** *147* (9), 7765-7776.

9. Fischer, M.; Georges, J., Fluorescence quantum yield of rhodamine 6G in ethanol as a function of concentration using thermal lens spectrometry. *Chem. Phys. Lett.* **1996,** *260* (1-2), 115-118.

10. Azuma, E.; Nakamura, N.; Kuramochi, K.; Sasamori, T.; Tokitoh, N.; Sagami, I.; Tsubaki, K., Exhaustive syntheses of naphthofluoresceins and their functions. *J. Org. Chem.* **2012,** *77* (7), 3492-500.

11. Liu, C.; Chen, W.; Shi, W.; Peng, B.; Zhao, Y.; Ma, H.; Xian, M., Rational design and bioimaging applications of highly selective fluorescence probes for hydrogen polysulfides. *J. Am. Chem. Soc.* **2014,** *136* (20), 7257-7260.

12. Chen, W.; Rosser, E. W.; Matsunaga, T.; Pacheco, A.; Akaike, T.; Xian, M., The development of fluorescent probes for visualizing intracellular hydrogen polysulfides. *Angew. Chem. Int. Ed.* **2015,** *127* (47), 14167-14171.

13. Ye, S.; Hananya, N.; Green, O.; Chen, H.; Zhao, A. Q.; Shen, J.; Shabat, D.; Yang, D., A highly selective and sensitive chemiluminescent probe for real-time monitoring of hydrogen peroxide in cells and animals. *Angew. Chem. Int. Ed.* **2020,** *59* (34), 14326-14330.

14. Hu, J. J.; Wong, N.-K.; Ye, S.; Chen, X.; Lu, M.-Y.; Zhao, A. Q.; Guo, Y.; Ma, A. C.-H.; Leung, A. Y.-H.; Shen, J., Fluorescent probe HKSOX-1 for imaging and detection of endogenous superoxide in live cells and in vivo. *J. Am. Chem. Soc.* **2015,** *137* (21), 6837-6843.
